# Forecasting compound sea level extremes: the 29–30 October 2018 Adriatic storm surge and meteotsunami extreme

**DOI:** 10.1007/s11069-026-08434-w

**Published:** 2026-10-10

**Authors:** Krešimir Ruić, Jadranka Šepić, Maja Telišman Prtenjak, Kristian Horvath, Marko Kvakić

**Affiliations:** 1 Faculty of Science, University of Split, Split, Croatia; 2 Department of Geophysics, Faculty of Science, University of Zagreb, Zagreb, Croatia; 3 Croatian Meteorological and Hydrological Service, Zagreb, Croatia

**Keywords:** Compound extreme, Storm surge, Meteotsunami, WRF, SCHISM, Early-warning

## Abstract

On 29–30 October 2018, an extreme sea level occurred in the Adriatic Sea, driven by a combination of long-period (storm surge) and short-period (meteotsunami) oscillations. Storm surges were strongest in the northern Adriatic, while shorter-period oscillations dominated in the middle Adriatic, where a meteotsunami was observed and recorded in Vela Luka Bay (Korčula Island). This type of extreme sea level event, arising from the combination of a storm surge and a meteotsunami, is termed a compound extreme and poses both a major threat and a forecasting challenge. Using this event as a case study, we evaluated two different approaches to forecasting compound sea level extremes. The first approach relies solely on numerical modelling: sea level response was modelled using the Semi-implicit Cross-scale Hydroscience Integrated System Model (SCHISM), forced by outputs from the Weather Research and Forecasting atmospheric numerical model (WRF). This approach reproduced the storm surge but failed to reproduce the shorter-period oscillations, including the observed meteotsunami. The second, hybrid, approach combines the first approach for modelling the storm surge component with the shorter-period sea level oscillations obtained from SCHISM forced by the high-frequency component of measured air pressure time series. As the meteotsunami was strongest in Vela Luka, the second approach was tested only at this location. With the second approach, both the storm surge and the magnitude, time of onset and duration of the meteotsunami were successfully reproduced. The results suggest that the second approach could provide basis for an early, site-specific warning system for compound extremes.

## Introduction

Extreme sea levels occur across a wide range of temporal and spatial scales, from global (thousands of kilometres) to local (hundreds of metres), and across periods ranging from centuries to minutes. Although the processes that generate extreme sea levels can occur independently, they may also combine to produce compound extremes, i.e. extremes which are caused by a combination of several processes which have different spatial and/or temporal scales. A compound extreme event is, in the context of this paper, defined as an extreme sea level event in which low-frequency (*T* > 2 h) and high-frequency (*T* < 2 h) sea level components contribute significantly to the overall extreme sea level. The threshold period is set at 2 h, as it roughly separates sea level extremes which are due to storm surges and other longer period processes from extremes which are due to shorter time scale processes. These shorter processes include: (i) infragravity waves with periods up to 300 s (Bertin et al. 2018), (ii) free and forced long ocean waves which most commonly have periods of tens of minutes, such as tsunamis and meteotsunamis (Pattiaratchi and Wijeratne 2015), and (iii) seiches (i.e., standing waves) with periods which are dependant on the geometry of the harbour, bay or shelf (Vilibić and Šepić 2017). Wind waves and swells are also short-period extremes, however as they occur on periods shorter than 1 min and are not captured by available tide gauge measurements and numerical models, they are not subject of our research, i.e., they are not included in compound event definition.

The primary driver of low-frequency sea level extremes is storm surge (Pugh and Woodworth 2014). Storm surges develop due to atmospheric pressure drop (and related inverse barometer effect), wind-driven water piling and wave setup. The inverse barometer effect occurs when a drop in atmospheric pressure over the ocean leads to a rise in sea level with sea level rising for approximately 1 cm for each 1 hPa of air pressure drop (Wunsch and Stammer 1997). In addition, intense low-pressure systems, such as those within tropical and extratropical cyclones, generate strong winds that can push water toward the coast, amplifying the surge (von Storch and Woth 2008) which, in combination with wave setup and runup, can result with hazardous inundation (Stockdon et al. 2006). The surges are most pronounced in places where the wind fetch is long enough for the wind-driven water pilling and wave setup and where deep low-pressure systems are common (von Storch and Woth 2008; Needham et al. 2015).

The primary driver of high-frequency (*T* < 2 h) sea level extremes are long ocean waves and seiches, which are generated through a variety of processes, including seismic and meteorological processes. In this paper, we focus on the latter. The largest of meteorologically triggered long ocean waves are known as meteorological tsunamis or meteotsunamis (Monserrat et al. 2006; Vilibić et al. 2025). Meteotsunamis are long ocean waves which are initiated by the resonant transfer of energy from the atmospheric pressure disturbances to the ocean through Proudman (Proudman 1929) or Greenspan resonance (Greenspan 1956). Apart from these resonant processes, a few more steps need to occur for the meteotsunami to reach dangerous heights. At the open coast, additional step includes amplification of long ocean waves due to nonlinear processes and shoaling near the coast. In bays and harbours three additional processes can happen: (i) further amplification within bays due to shoaling and progressive narrowing of a bay from its wide open mouth towards its narrow enclosed end; (ii) wave reflection at the closed end of the bay, resulting in further amplification; and (iii) harbour resonance, occurring when waves reflected from the closed end of the bay constructively interfere with the incoming long waves (Orlić et al. 2010). Because of all the required processes, strong meteotsunamis usually happen in specific places, called meteotsunami hotspots, such as the Balearic Islands in Spain, the Baltic and Black Sea, the Great Lakes, Nagasaki Bay in Japan, the Adriatic Sea, and others (Monserrat et al. 2006; Rabinovich 2020; Vilibić et al. 2021).

There are a lot of case studies of storm surges worldwide and a lot of separate studies of meteotsunamis, especially for the hotspot locations. However, there are considerably fewer studies focusing on the compound extremes, i.e., on sea level extremes that are both due to low and high-frequency sea level oscillations. Selected papers that deal with compound extremes included case studies on storms Gudrun and Gloria (Suursaar et al. 2006; Pérez-Gómez et al. 2021), typhoons Lionrock and Jebi, Maysak and Songda (Heidarzadeh and Rabinovich 2021; Medvedev et al. 2022; Rabinovich et al. 2023). Furthermore, systematic approaches for extracting and analysing compound extremes from multi-year sea level time series have recently been developed by Ruić et al. (2023) for the Adriatic Sea and by Ramos-Alcántara et al. (2025) for the Balearic Islands.

The focus of this paper is the Adriatic Sea, in which both storm surges and meteotsunamis happen regularly (Vilibić et al. 2017; Šepić and Orlić 2024). The Adriatic Sea storm surges occur in the presence of an extratropical cyclone (cyclone from now on), usually one originating from the western Mediterranean (gulf of Genoa) and crossing over the Adriatic: the cyclone raises the Adriatic sea level through inverse barometric effect and water piling due to strong southeasterly Sirocco winds (Robinson et al. 1973; Lionello et al. 2012). On the other hand, the Adriatic Sea meteotsunamis occur due to mesoscale atmospheric disturbances (e.g., long-lasting convection or ducted gravity waves; Belušić and Strelec Mahović 2009; Vilibić and Šepić 2009), and these mesoscale atmospheric disturbances commonly appear under synoptic conditions that include: (i) low surface air pressure to the west of the affected area; (ii) an inflow of warm, dry air from the southwest in the lower troposphere (commonly reported for an altitude of 850 hPa); (iii) strong southwesterly winds at the mid and upper troposphere (commonly reported for an altitude of 500 hPa); and (iv) the presence of unstable atmospheric layers, characterized by a low Richardson number, *Ri* < 0.25, at mid troposphere (~ 500 hPa) (Vilibić and Šepić 2009; Šepić et al. 2015a). Similar synoptic conditions have been associated with meteorological tsunamis in other parts of the Mediterranean (Ramis and Jansà 1983). Although this is the most common tsunamigenic synoptic weather pattern over the Mediterranean, it has also been shown that meteotsunamis can occur jointly with storm surges, i.e., when cyclones impact the Adriatic Sea (Ferrarin et al. 2021; Šepić and Orlić 2024; Ruić et al. 2025). The Adriatic Sea meteotsunamis can also occur during bora wind episodes (Šepić and Orlić 2024; Ruić et al. 2025).

Although there are many case studies of the Adriatic Sea storm surges, as well as many case studies of meteotsunamis, the joint (compound) extremes have mostly been overlooked. One example of the Adriatic Sea compound flooding extreme is the Venice flood of 12 November 2019, during which a sea level of 189 cm above the reference zero at Punta della Salute tide gauge was recorded (Ferrarin et al. 2021). According to Orlić and Pasarić (2024) the storm surge, defined in their paper as sea level variability at periods from 10 h to 10 days, contributed with 24.9% (47 cm) to this flood; meteotsunami (periods from 2 min to 10 h) with 14.8% (28 cm); and tidal oscillations and remaining sea level components (with periods longer than 10 days), together accounted for 60.3% (114 cm). Although the contributions of surge and meteotsunami may seem small, jointly, for Venice, combination of the two-represents a major change in the percentage of the flooded parts of the city. A sea level of 110 cm floods 12% of the historic city core, while a level of 140 cm floods 59%, and a sea level of 189 cm almost 90% (Anzidei et al. 2024).

Mitigation of the potential damage that compound extreme can cause is possible via a forecast system. For the Adriatic Sea, there are at least two operational systems for storm surge prediction which are focused on the northern Adriatic and Venice (Umgiesser et al. 2021; Rus et al. 2023, 2025). Since storm surge is the result of synoptic processes that are evident on larger spatial scales (~ 100 km) and are successfully predicted by numerical atmospheric models, storm surge forecasts are rather reliable. On the other hand, meteotsunamis are generated by small scale and mesoscale atmospheric processes which are not easily reproduced by atmospheric forecast model and thus meteotsunamis are not readily forecasted. The only currently operative meteotsunami forecast and warning system is one for the Balearic Islands operated by the Spanish State Meteorological Agency (AEMET) (Jansà and Ramis 2021). The system has initially been based on assessment of synoptic conditions but is now also supplemented by the analysis of high-resolution ocean numerical models (Mourre et al. 2021; Romero 2024).

Currently, the forecast of compound extremes is virtually non-existent, and even the post reproduction of such events through numerical modelling is still in its early stages (Kim and Omira 2024). Considering this, the purpose of our paper is to investigates the potential for forecasting compound extremes, using the Adriatic Sea compound event of 29–30 October 2018 as a case study and further focusing on the Vela Luka Bay for a target of the compound extreme prediction model. The primary goal is to test the method for a location at which both processes occur simultaneously and induce flooding, such as Vela Luka Bay (Orlić et al. 2010; Šepić and Orlić 2024). This methodology can be used in future work to expand the prediction to various other locations.

The compound extreme event of 29–30 October 2018 manifested as storm surges in the northern Adriatic (Cavaleri et al. 2019; Morucci et al. 2020) and as both storm surges and pronounced high-frequency sea level oscillations, including the Vela Luka meteotsunami, in the middle Adriatic (Šepić and Orlić 2024). As both meteotsunamis and storm surges are generated by atmospheric processes, the first attempt to forecast the 29–30 October 2018 compound extreme relied on using a numerical atmospheric model to reproduce the relevant atmospheric processes and then using its output to force an oceanographic numerical model. The Weather Research and Forecasting (WRF) model (Skamarock et al. 2008) was used for the atmospheric modelling and the Semi-implicit Cross-scale Hydroscience Integrated System Model (SCHISM) (Zhang et al. 2016) for the ocean modelling. As storm surges occurred throughout the Adriatic, modelled sea level time series were compared with all available sea level measurements from tide gauges on the eastern and western Adriatic coasts. This first approach failed to capture the full magnitude and timing of the compound extreme, primarily because of the poor reproduction of the high-frequency sea level oscillations. We therefore tested another approach, which combined the low-frequency component of sea level oscillations (*T* > 2 h) obtained using the first approach with a high-frequency sea level series (*T* < 2 h) obtained using a hybrid approach. Here, the hybrid approach refers to forcing the numerical ocean model with high-frequency air pressure measurements. The second approach focused on Vela Luka Bay, i.e. on one location where both a surge and a strong meteotsunami were recorded. Outputs from both approaches are analysed in detail and forecast potential is discussed.

The paper is structured as follows: Sect.  2 brings Data and methods including the description of numerical models, Sect.  3 Extreme event analysis, Sect.  4 Event forecasting methods and Sect.  5 Discussion and conclusions.

## Data and methods

### Data collection and analysis

**Fig. 1:**
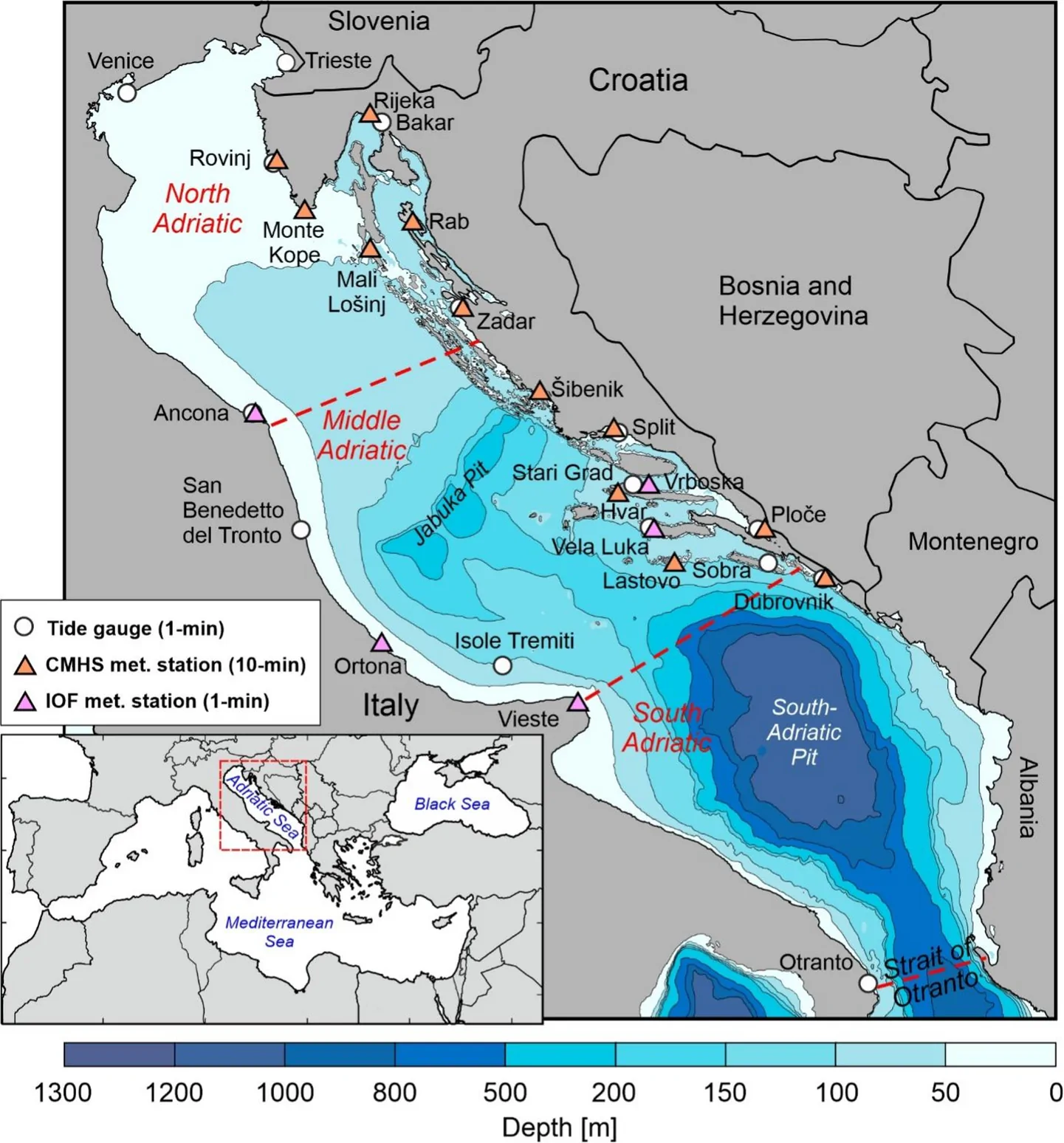
Locations of tide gauge stations (white circles) and meteorological stations from the CMHS (orange triangles; 10-min sampling rate) and the IOF (pink triangles; 1-min sampling rate). Names of all stations are given. Meteorological station Sv. Ivan na Pučini, which is close to Rovinj, is called Rovinj for simplicity

Sea level time series, measured with a 1-minute sampling rate, originating from 14 Adriatic tide gauges, locations of which are shown in Fig. 1, were used to analyse the 29–30 October 2018 compound sea level extreme. For stations Venice, Trieste, San Benedetto del Tronto, Ancona, Stari Grad, Vela Luka, Sobra and Otranto, data, originally measured by the national providers Istituto Superiore per la Protezione e la Ricerca Ambientale (ISPRA, Italy) and the Institute of Oceanography and Fisheries (IOF, Croatia), were gathered via IOC s Sea Level Station Monitoring Facility (SLSMF) web portal (https://www.ioc-sealevelmonitoring.org/, last access 24 June 2026). Data from Bakar were obtained from the Department of Geophysics at the Faculty of science (Zagreb, Croatia) and the time series from the remaining five tide gauges (Rovinj, Zadar, Split, Ploče and Dubrovnik) were obtained from the Hydrographic Institute of the Republic of Croatia (Split, Croatia). All sea level data used in the analysis were thoroughly visually checked and all spikes and non-physical oscillations were removed. Sea level series were de-tided using 7 harmonic constituents (K1, O1, P1, K2, S2, M2 and N2), which were shown by earlier research to be the most dominant tidal harmonics in the Adriatic Sea (Pasarić and Orlić 2001; Medvedev et al. 2020). The de-tiding was done using T-Tide package in MATLAB (Pawlowicz et al. 2002). Finally, residual sea level series were filtered using a 2 h Kaiser-Bessel window (Thomson and Emery 2024) into a low-frequency (*T* > 2 h) and high-frequency (*T* < 2 h) components. To determine periods of dominant oscillations, spectral analysis on the residual time series was performed both for the event and background (calm) periods. The event power spectrum density was calculated over period of 2048 points/minutes (~ 34 h) cantered around the event while background (calm) power spectrum density was calculated over calm (without significant sea level oscillations) period of 5120 points/minutes (~ 85 h). Both spectra were calculated using half-overlapping windows of 512 points length (~ 8.5 h).

Atmospheric pressure records, which were measured with a 1-minute sampling rate by the Institute of Oceanography and Fisheries in Split (IOF), include the following stations: Ancona, Ortona, Vieste, Vela Luka and Vrboska. The 10-minute sampling rate atmospheric pressure records which were measured by the Croatian Meteorological and Hydrological Service (CMHS) include data from: Dubrovnik, Ploče, Lastovo, Hvar, Split, Šibenik, Zadar, Mali Lošinj, Rab, Monte Kope, Rijeka and Sv. Ivan na Pučini (Rovinj). Locations of the IOF and CMHS pressure stations are shown in Fig. 1. Similarly to the sea level time series, atmospheric pressure time series were thoroughly checked, and subsequently filtered into a low-frequency (*T* > 2 h) and high-frequency (*T* < 2 h) components using Kaiser-Bessel window with a cut-off period of 2 h.

**Fig. 2:**
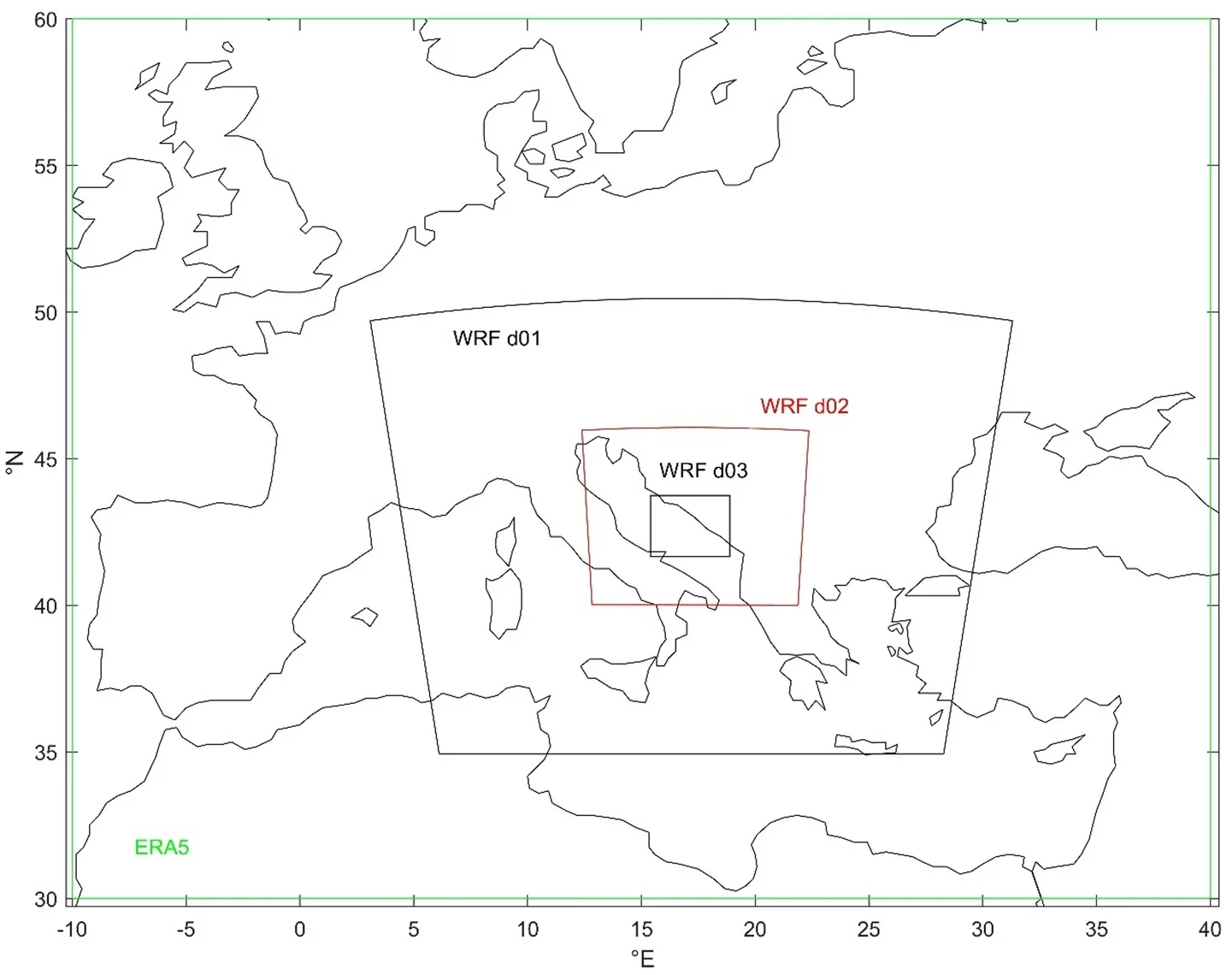
The ERA5 domain (used in the manuscript) and three WRF domains: outputs of the d02 domain simulations were used to force the ocean model (SCHISM)

Relevant synoptic data including: (i) mean sea level pressure, *u* and *v* component of wind speed at 10 m; (ii) temperature and *u* and *v* component of wind at 850 hPa height; and (iii) geopotential height of 500 hPa, and *u* and *v* component of wind at 500 hPa height, were obtained from the ERA5 reanalysis (Copernicus Climate Change Service, Climate Data Store 2023; Hersbach et al. 2023). ERA5 variables were downloaded over a domain indicated in Fig. 2.

### WRF numerical model setup

Atmospheric numerical modelling was performed using the Weather Research and Forecasting (WRF) model (Skamarock et al. 2008). Three two-way nested model domains, cantered over the Adriatic Sea, were selected (Fig. 2). The largest domain (d01) encompasses a wide area stretching from ~ 35 to ~ 50°N in meridional direction, and from ~ 5 to ~ 30°E in zonal direction. The intermediate domain (d02) covers most of the Adriatic Sea, and the innermost domain is located in the central Adriatic. The horizontal grid spacing is 9 km in d01 (228 × 187 point grid), 3 km in d02 (259 × 223 grid), and 1 km in domain d03 (292 × 235 grid), maintaining a spatial and temporal nesting ratio of 1:3. The vertical grid, using terrain-following coordinates, consists of 97 vertical levels resolving the atmosphere up to 50 hPa. The density of vertical levels decreases toward the top of the model domain. For the purposes of forecasting efforts presented in this study, data from the intermediate domain (d02) were used to force the ocean SCHISM model.

The physical parameterizations employed include: (i) the WSM6 (WRF-Single-Moment 6-class) microphysics scheme (Hong and Lim 2006), which accounts for ice crystals, snow, and graupel in high-resolution simulations; (ii) the RRTM scheme for longwave and shortwave radiation processes (Mlawer et al. 1997); (iii) the Mellor-Yamada Nakanishi Niino (MYNN) scheme for the planetary boundary layer (Nakanishi and Niino 2006); and (iv) the Noah–MP land surface model (Niu et al. 2011). The Kain-Fritsch cumulus parameterization (Kain 2004) was used only in the outermost domain. The urban surface processes were represented using the Urban Canopy Model scheme (Chen et al. 2011) and additional options were selected within the model to account for sloped terrain effects (Zängl 2002). Initial and boundary conditions (updated every 6 h) were derived from the ERA5 reanalysis dataset at approximately 10 km resolution, starting on 28 October 2018 at 12:00 UTC. Topographic data were loaded at a resolution of 900 m and combined with MODIS satellite land cover data at a 450 m resolution, available from the following website (last accessed on 23 December 2025): https://www2.mmm.ucar.edu/wrf/users/download/get_sources_wps_geog.html#specific.

### SCHISM numerical model setup

Oceanographic numerical modelling was conducted using the Semi-implicit Cross-scale Hydroscience Integrated System Model (SCHISM). SCHISM is an unstructured, three-dimensional baroclinic numerical model (Zhang et al. 2016). To model extreme sea levels, an option for using two-dimensional barotropic model based on the shallow water equations can be chosen. The two-dimensional model, which we have used, solves the following equations:

1 $$ \frac{{\partial \eta }}{{\partial t}} + \nabla \cdot \left( {\left( {h + \eta } \right)\boldsymbol{U}} \right) = 0, $$

2 $$\:\frac{\partial\:\boldsymbol{U}}{\partial\:t}+\boldsymbol{U}\nabla\:\boldsymbol{U}=\:-\nabla\:\left(\frac{{p}_{a}}{\rho\:}+g\eta\:\right)-f\boldsymbol{k}\:\times\:\boldsymbol{U}+\frac{{\boldsymbol{\tau\:}}_{\boldsymbol{w}}-{C}_{b}\rho\:\left|\boldsymbol{U}\right|\boldsymbol{U}}{\rho\:\left(h+\eta\:\right)}+{\boldsymbol{F}}_{\boldsymbol{h}},$$

where *t* is time, *h* is depth (defined by bathymetry, positive from mean sea level downward), *g* is gravitational acceleration, *x* and *y* are Cartesian coordinates, ***U*** is the vertically integrated current velocity, *η* is sea surface elevation, *pₐ* is atmospheric pressure, *ρ* is seawater density, *f* is the Coriolis parameter, ***k*** is the unit vector pointing upward, $\:{\boldsymbol{\tau\:}}_{\boldsymbol{w}}$ is the wind stress vector, $\:{\boldsymbol{F}}_{\boldsymbol{h}}$ is the horizontal diffusion vector, and $\:{C}_{b}$ is the bottom friction coefficient (set to 0.003) (Zhang 2014). Tidal oscillations were not included in the model.

**Fig. 3:**
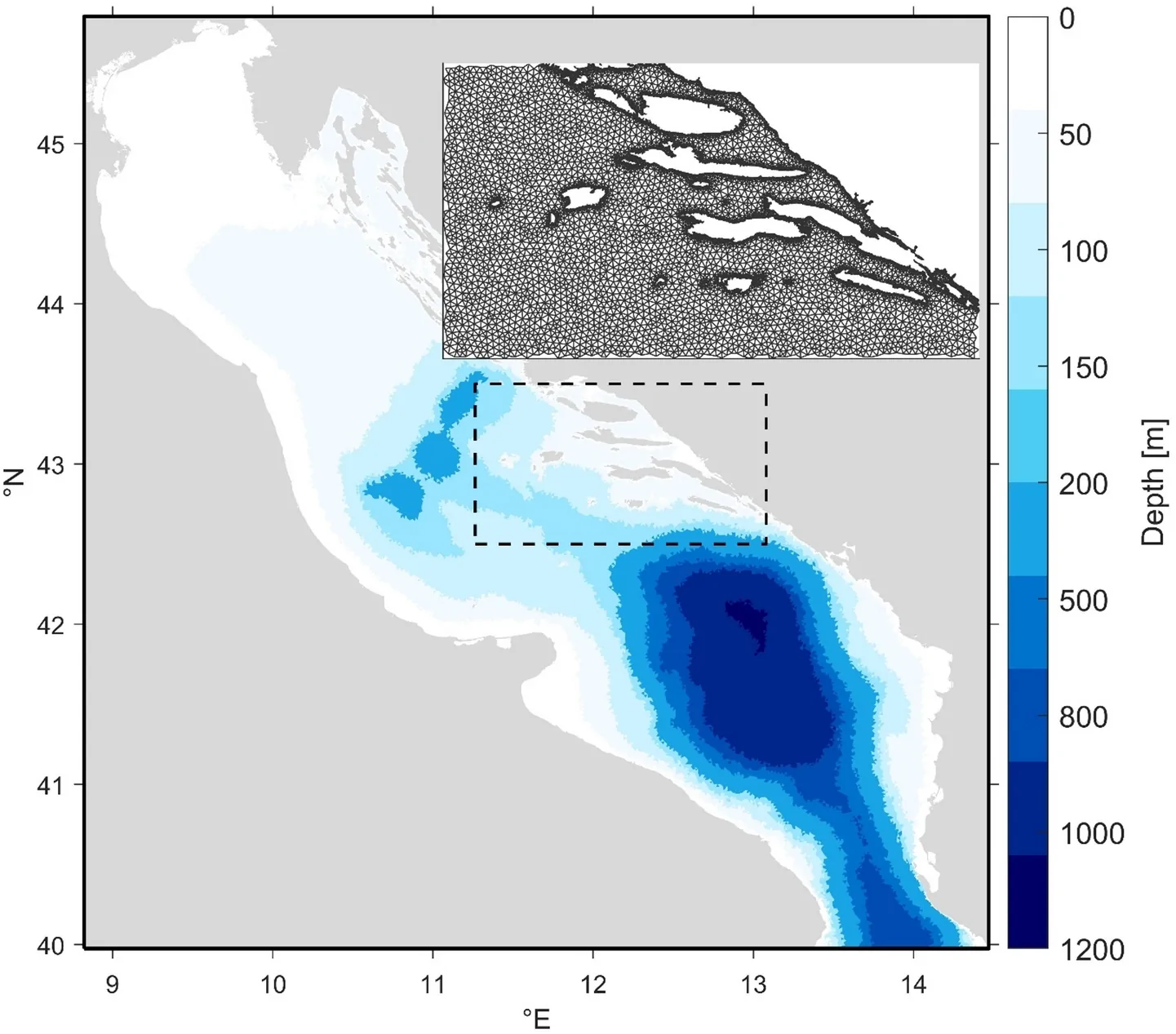
View of the SCHISM domain: the larger image shows the SCHISM model bathymetry, while the inset shows the SCHISM mesh elements for the area marked by the rectangle in the larger image

The model domain covers the entire Adriatic Sea and consists of 1,34,065 elements and 84,280 nodes. The density of the domain elements is higher near the coastline and lower in the open sea, as can be seen in Fig. 3 where a part of the domain is shown. Boundary conditions were prescribed at the Strait of Otranto. A constant value (zero) was used for sea level elevation at the open boundary, while the radiative boundary condition was applied for currents. The model time step was set to one minute to account for the Courant criterion (Zhang 2014).

The skill of the numerical model was examined by using root mean square error (*RMSE*) and *Bias*. *RMSE* was calculated as:

$$\:RMSE=\sqrt{\frac{1}{n}\sum\:_{i=1}^{n}{\left({y}_{i}-{x}_{i}\right)}^{2}},$$

where modelled values are denoted by * y* and measured values by * x*, and *n* is number of points for which comparison was made.

*Bias* was calculated, using the same naming conventions as:

$$\:Bias=\frac{1}{n}\:\sum\:_{i=1}^{n}({y}_{i}-{x}_{i}).$$

## Compound sea level extreme of 29–30 October 2018

The extreme sea level event which occurred on 29–30 October, 2018, is partly described on the online pages of the Adriatic meteotsunami catalogue (Šepić and Orlić 2024), on the official website of the City of Venice, which contains descriptions of the 20 strongest extreme sea level events recorded at Venice tide gauge Punta della Salute, starting with the 16 April 1936 event and ending with the 12 November 2019 event (https://www.comune.venezia.it/it/content/le-acque-alte-eccezionali#29ottobre, last accessed (3 July 2026), and in numerous newspaper articles. Šepić and Orlić (2024) describe meteotsunami which occurred in Vela Luka (Korčula island, Fig. 1); official website of the City of Venice describes the surge in Venice, and newspaper articles mostly describe floodings and damages due to both meteotsunami and surge along the eastern (Croatian) Adriatic coast.

### Synoptic situation

**Fig. 4:**
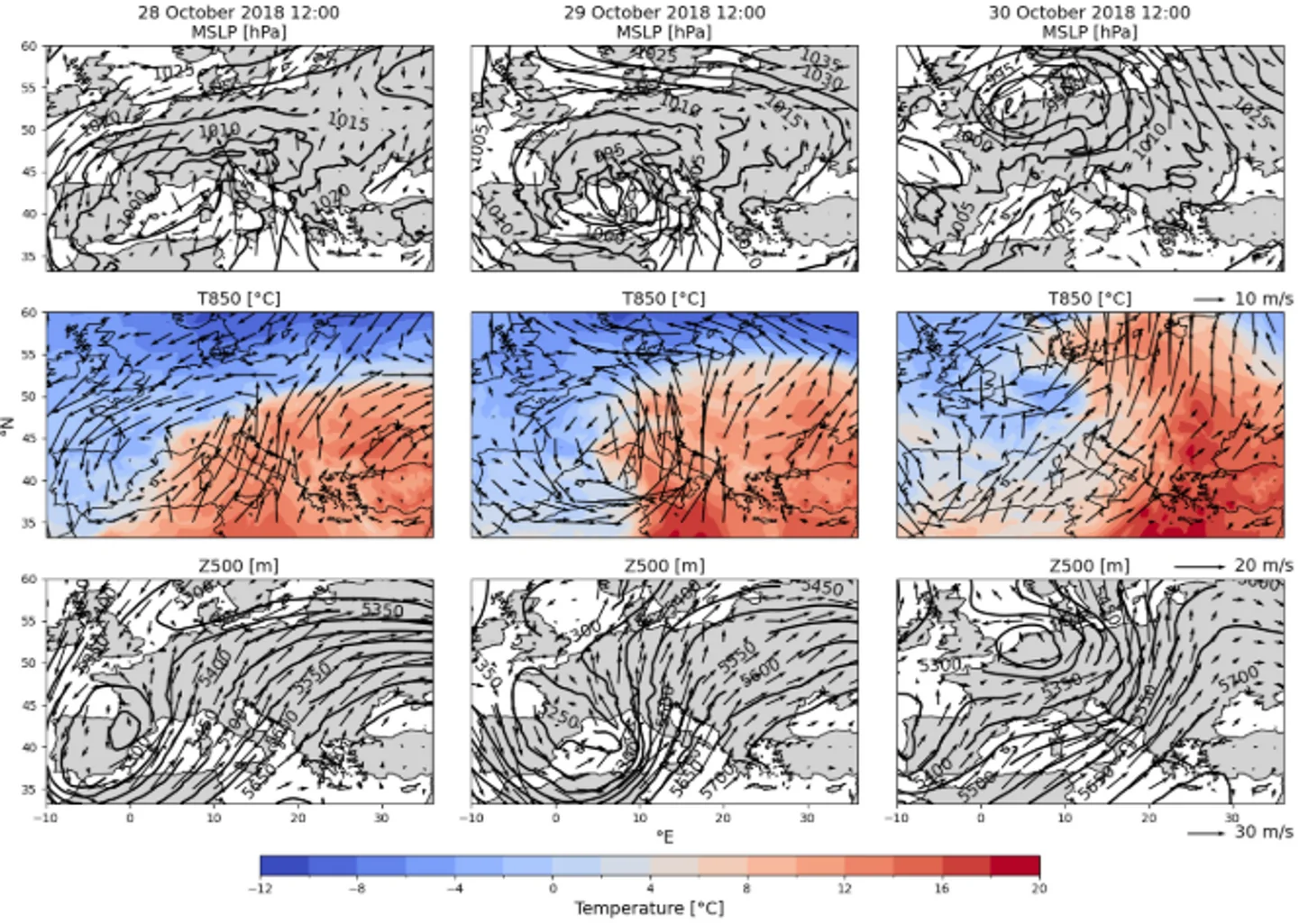
Mean sea level pressure (top row), temperature at 850 hPa (middle row), and geopotential height at 500 hPa (bottom row) for the three analysed dates. The first column shows the synoptic situation on 28 October 2018, the second on 29 October 2018, and the third on 30 October 2018 (all at 12:00 UTC). Wind vectors are shown at each pressure level. Wind vectors are scaled differently at each pressure level, with corresponding reference vectors indicated in the bottom-right corner of each row

The synoptic background of the event was analysed using ERA5 reanalysis data, as shown in Fig. 4 for 28, 29 and 30 October (at 12:00 UTC for all days). For each date, the figure displays mean sea level pressure (MSLP) fields (top row), temperature at the 850-hPa level (second row; T850), and geopotential height of the 500-hPa plane (third row; Z500). Wind vectors are shown for all levels. The selected fields are those identified by earlier research to be representative of synoptic situations associated to meteotsunami and storm surge generation (Vilibić and Šepić 2009; Lionello et al. 2012; Šepić et al. 2015a) and are also fields which are commonly used to assess a synoptic situation.

On 28 October at 12:00 UTC (first column of Fig. 3), an extratropical cyclone was located over the western Mediterranean. Associated with it, pronounced pressure gradients developed over the Adriatic Sea, resulting in southeasterly Sirocco winds (up to 15 m/s) that transported seawater towards the northern Adriatic. At the 850-hPa level, warm air advection from Africa was present across much of the Mediterranean, peaking over the Central Mediterranean and the Adriatic Sea. Winds over the Adriatic at this level were southwesterly and reached speeds of up to approximately 20 m/s. The pattern of geopotential height of the 500-hPa level reveals that an upper-level closed low was positioned over Spain, with strong southwesterly winds (up to 35 m/s) blowing across much of the Mediterranean, from the Balearic Islands to the Ionian Sea. The vertical tilt between the surface cyclone centre and the centre of the upper-level low suggests that at this stage cyclone is deepening and converting potential energy of the atmosphere to kinetic energy of the cyclone through baroclinic instability.

On the following day, 29 October at 12:00 UTC, the centre of the extratropical cyclone intensified and moved towards the Gulf of Genoa. Mean sea level pressure over the Adriatic Sea ranged from 990 hPa in the north to 1015 hPa in the south, producing strong west-east pressure gradients and triggering gale-force southeasterly winds (up to 20 m/s) across the entire Adriatic. The winds continued to push seawater northward. The warm air advection pattern at the 850-hPa level shifted slightly eastward, with strong southerly winds over the central Mediterranean and the Adriatic Sea reaching speeds up to 25 m/s, causing a pronounced warm air advection ($\:{T}_{a}\:>16\:^\circ\:C$) from the southern Mediterranean and Africa. The centre of the closed low at 500 hPa moved eastward and was now located over the western Mediterranean Sea. The tightening of the 500-hPa isohypses indicated a strengthening of the southwesterly jet over the Adriatic, with mid-tropospheric winds reaching speeds up to 40 m/s.

Finally, by 12:00 UTC on 30 October, the surface cyclone had passed over the Adriatic, resulting in a weakening of pressure gradients and southeasterly winds over the Adriatic Sea (down to ~ 10 m/s). The 850-hPa warm air advection field had shifted further eastward (centred over Greece), resulting with cooler air masses positioned over the northern Adriatic ($\:{T}_{a}\:\sim2\:^\circ\:C$) and warmer over the southern Adriatic ($\:{T}_{a}\:\sim8\:^\circ\:C$). The 500-hPa geopotential height pattern continued to favour strong southwesterly winds (up to 30 m/s) over the Adriatic Sea.

The intensification of surface southeasterly winds over the Adriatic, along with the presence of a low pressure system (extratropical cyclone), represents a typical setting for the development of the Adriatic storm surges (Robinson et al. 1973; Lionello et al. 2012). The intrusion of warm air at 850 hPa from the south, in combination with a strong south to southwesterly jet stream at 500 hPa, are, on the other hand, additional synoptic characteristics recognized as favourable for the development of extreme high-frequency sea level oscillations, including Adriatic meteotsunamis (Vilibić and Šepić 2009; Šepić et al. 2015a; Šepić and Orlić 2024; Ruić et al. 2025).

### Meteorological measurements

**Fig. 5:**
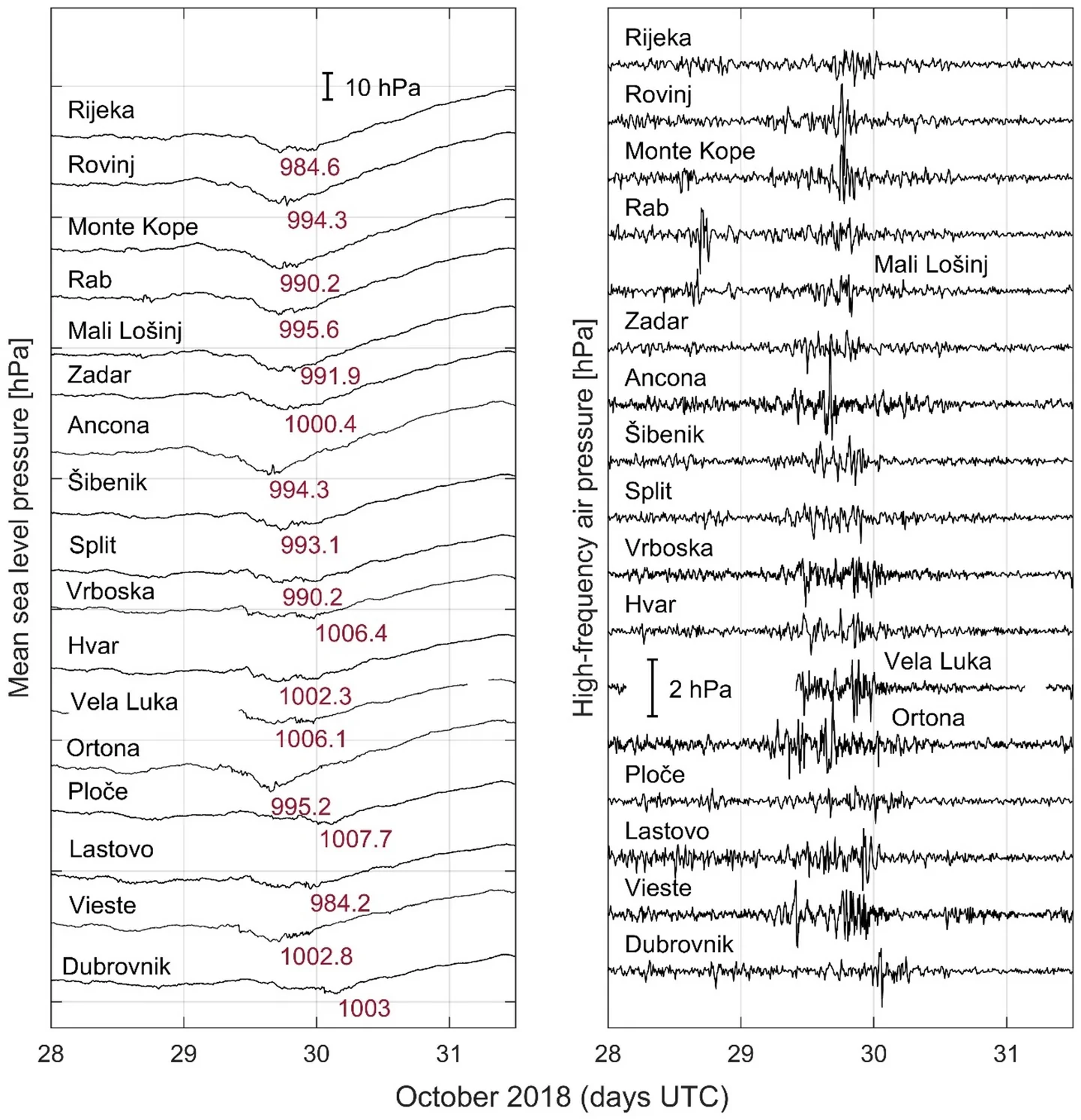
Left: measured mean sea level pressure time series; Right: high-frequency (*T* < 2 h) component of air pressure for available meteorological stations (see Fig. 1 for locations). Series are vertically offset for clearer presentation. Values of minimum MSLP measured at each station are given with red letters

Original and high-frequency (*T* < 2 h) air pressure time series, from stations whose locations are given in Fig. 1, are shown in Fig. 5. All stations exhibit a pronounced drop in air pressure, with the timing of the minimum pressure approximately coinciding with the timing of the sea level extremes recorded at tide gauge stations (between 18:00 UTC on 29 October and 02:00 UTC on 30 October). Values of the pressure drop within a 12-hour interval preceding the recorded air pressure minimum are given in Table 1. The largest air pressure drop for the northern Adriatic stations was recorded at Rovinj, amounting to 10.5 hPa/12 h; for the central Adriatic, the largest drop was detected at Ortona (11.12 hPa/12 h); and for the southern Adriatic stations, the largest drop, 8.3 hPa/12 h, was recorded at Lastovo (Table 1).

As for the high-frequency air pressure oscillations, a significant intensification was first detected at the northern Adriatic stations Rab and Mali Lošinj on 28 October. During the following day, high-frequency air pressure oscillations intensified at the remaining northern Adriatic stations as well, peaking between 18:00 and 22:30 UTC on 29 October, with strongest oscillations recorded at Rovinj and Monte Kope (absolute pressure change rates of 2.0 and 1.4 hPa per 10 min, respectively).

In the middle Adriatic, intensification of high-frequency air pressure oscillations was most pronounced between 13:00 UTC of 29 October and 01:00 UTC of 30 October, with high-frequency oscillations intensifying at more northern stations (Ancona and Zadar) earlier (between 13:00 and 17:00 UTC) and at more southern stations (e.g. Ortona) later (early hours of 30 October). During this period, pronounced high-frequency pressure oscillations were recorded at almost all central Adriatic stations, with the highest rate of air pressure change, 1.57 hPa per 10 min, detected at Vela Luka (Table 1).

For the southernmost Adriatic stations (Dubrovnik and Vieste), the onset of pronounced high-frequency air pressure oscillations was observed at the later time, i.e., between 00:00 and 05:00 UTC on 30 October.

Onset times of pronounced high-frequency air pressure oscillations, with oscillations starting at the northern Adriatic on 28 October, and spreading to middle (afternoon hours of 29 October) and southern (early morning hours of 30 October) Adriatic, suggests that atmospheric conditions favourable for triggering high-frequency air pressure oscillations propagated eastward during these three days.

**Table 1:** List of meteorological stations with the time of recorded minimum air pressure and its values; the magnitude of maximum pressure drop within the 12-hour period preceding the minimum; and the timing and value of the maximum 10-minute rate of change of the high-frequency component of air pressure

Measuring station	Time of minimum air pressure	Minimum air pressure [hPa]	Maximum air pressure drop [hPa/12 h]	Time of maximum HF oscillations (hh: mm UTC)	Maximum rate of HF air pressure change [hPa/10 min]
Rijeka	17:20	984.6	6.9	20:50	1.02
Rovinj	18:40	994.3	10.5	22:10	1.99
Monte Kope	17:20	990.2	8.8	21:30	−1.4
Rab	17:10	995.6	7.8	**18:40	1.18
Mali Lošinj	20:00	991.9	10.1	21:30	−1.12
Zadar	18:00	1000.4	6.6	13:50	−1.14
Ancona	15:24	994.3	10.4	16:22	−0.47
Šibenik	17:30	993.1	6.9	18:30	1.12
Split	17:50	990.2	5.3	15:00	−1.27
Vrboska	23:34	1006.4	7.8	20:24	−0.49
Hvar	17:00	1002.3	5.9	16:10	1.29
Vela Luka	16:18	1006.1	5.6	21:10	1.57
Ortona	15:42	995.2	11.1	*00:56	0.5
Ploče	*00:20	1007.7	7.2	*00:10	−0.84
Lastovo	22:50	984.2	8.3	*01:30	1.46
Vieste	17:13	1002.8	7.9	20:00	−0.56
Dubrovnik	*03:40	1003.0	7.4	*05:00	1.22

The date of the presented time is the 29 October 2018 for all station except for the ones where there is an asterisk (*), in which case the date is 30 October 2018 and in the case of a double asterisk (**) the date is 28 October 2018

### Sea level measurements

Ruić et al. (2023) extracted residual and high-frequency sea level extremes from 1-minute sea level time series measured from 2003 (at the earliest) to 2021 at 18 tide gauge stations spread evenly across the eastern and the western coast of the Adriatic Sea. Residual extremes were defined as intervals in which residual sea level height surpassed its 99.85 percentile threshold (different at each station), and high-frequency extremes as intervals in which high-frequency sea level height surpassed its 99.993 percentile (also different at each station). During 29–30 October 2018, 14 of the above-mentioned 18 tide gauges were operational. At 9 tide gauges both residual and high-frequency extremes were recorded, accompanied with the increase in the low-frequency sea level (larger than the 95 percentile), classifying the event as a compound extreme. At the southernmost station, Otranto, the event was not captured as extreme in neither of the time series (residual and high-frequency) while at Dubrovnik the event was only a high-frequency extreme, and at stations Zadar, Venice and Ancona the event was extreme only in the residual time series. Values of the residual and high-frequency maximum values, as well as their time of occurrence, are presented in Table 2 for all stations.

At all stations where the event was recognized as extreme, the maximum of the residual signal was recorded between 18:00 UTC on 29 October and 02:00 UTC on 30 October. At nearly all stations the maximum of the high-frequency signal occurred simultaneously with the residual maximum, implying that total maxima were reached due to superposition of low-frequency and high-frequency signal. Exceptions were Ploče and San Benedetto del Tronto, where the high-frequency maxima lagged behind the residual maxima by 2.5–3 h (Table 2).

**Table 2:** List of the tide gauge stations with the times of the residual maximum and the time lag of the high-frequency maximum (only for stations at which high-frequency extreme occurred) relative to the residual maximum

Tide gauge	Time of residual maximum (date, hh: mm UTC)	Value of the residual maximum [cm]	Delay of the HF maximum (hh: mm UTC)	Value of the HF maximum [cm]
Otranto	*2:38	15.7	0	11.5
Dubrovnik	*1:34	42.0	0	**6.7**
Sobra	*1:18	**58.6**	0	**21.9**
Ploče	23:35	**60**,**0**	+ 02:59	**19.9**
Vela Luka	20:32	**79.7**	0	**46.9**
Stari Grad	18:47	**61.5**	0	**25.9**
Split	*0:49	**60.6**	0	**11.8**
Zadar	23:06	**77.7**	−	9.0
Bakar	21:25	**133.9**	0	**30.0**
Rovinj	19:47	**116.7**	0	**12.0**
Trieste	18:47	**146.0**	0	**14.9**
Venice	19:09	**152.0**	−	9.0
Ancona	22:05	**78.5**	−	11.1
S. B. del Tronto	22:52	**69.6**	+ 02:36	**22.8**

Additionally, the values of the residual and high-frequency maxima are given. Residual and HF maxima recognized as extreme Adriatic sea level events, following methodology by Ruić et al. (2023), are bolded. The date of the presented time is the 29 October 2018 for all station except for the ones where there is an asterisk (*), in which case the date is 30 October 2018

**Fig. 6:**
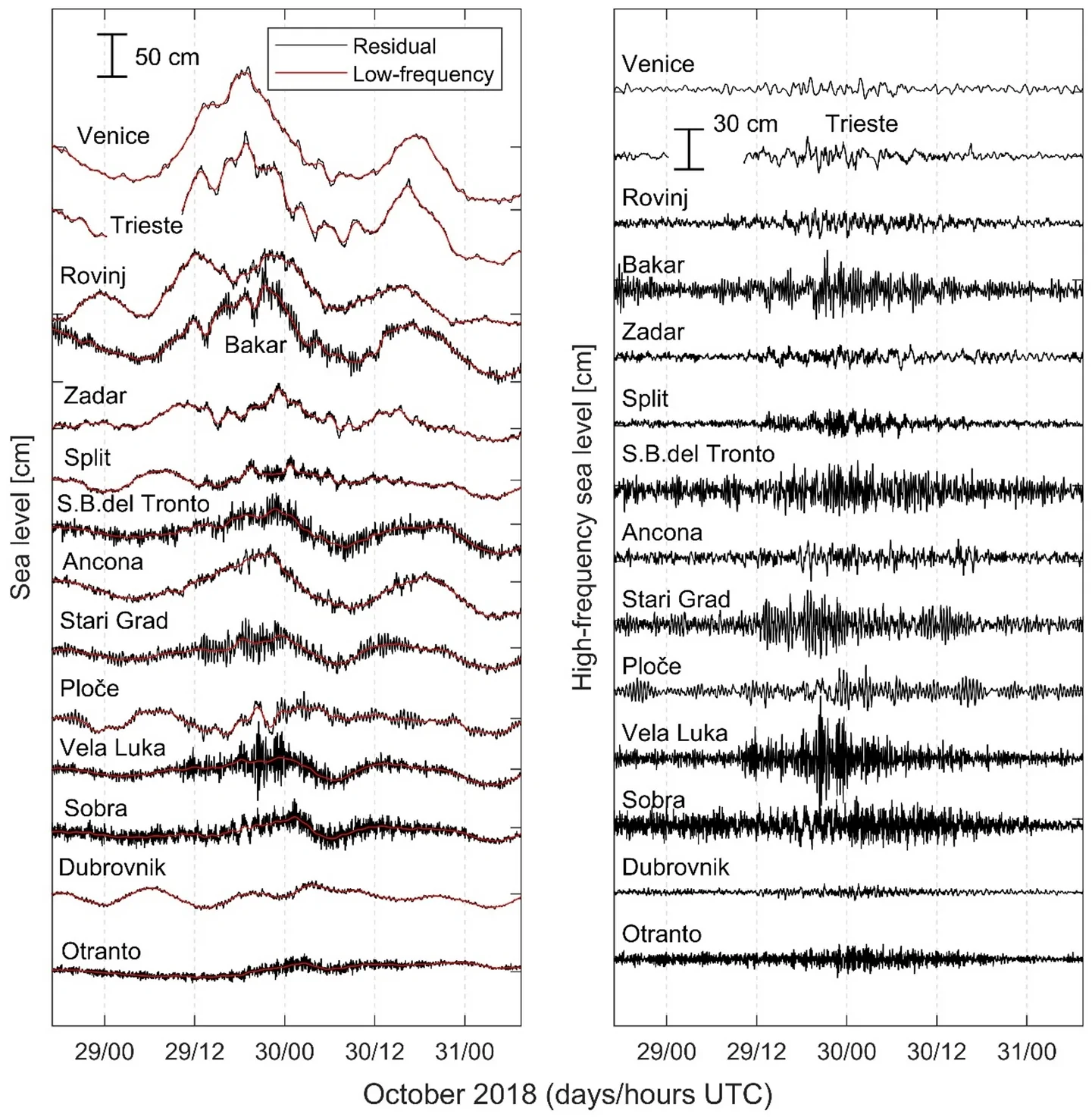
Left: residual (black line) and low-frequency (*T* > 2 h, red line) sea level time series; Right: high-frequency (*T* < 2 h) sea level time series (see Fig. 1 for locations of tide gauge stations). Series are vertically offset for clearer presentation

Residual, low-frequency (*T* > 2 h) and high-frequency (*T* < 2 h) sea level time series from 14 Adriatic tide gauge stations for the period from 29 October to 1 November 2018 are shown in Fig. 6. The maximum of the residual signal occurred during the late hours of 29 October and the early hours of 30 October, coinciding with the timing of the minimum sea level pressure over the Adriatic Sea (Fig. 5). The highest values of the residual signal, 152.0 cm and 146.0 cm (residual signal is given with respect to a station-specific sea level average which was estimated over the entire period for which 1-minute sea level time series were available), were recorded in Venice and Trieste, respectively (Fig. 6; Table 2). Moving southward, maximum values decrease, reaching 69.6 cm at San Benedetto del Tronto in the central Adriatic, and 58.6 cm at Sobra in the southern Adriatic.

At all stations, from Venice in the north to Otranto in the south, an intensification of high-frequency sea level oscillations was observed simultaneously to the rise of the residual signal. High-frequency oscillations ranged from 6.7 cm at Dubrovnik to 46.9 cm at Vela Luka (Table 2). In Vela Luka the high-frequency maximum was due to a meteotsunami (https://projekti.pmfst.unist.hr/floods/meteotsunamis/27-vela-luka-29-october-2018/, last access: December 2, 2025). Notably, the highest values of the high-frequency component occurred predominantly at stations in the middle Adriatic. These stations were described by Ruić et al. (2023) as the stations at which high-frequency oscillations significantly contribute to extreme sea levels. Pronounced high-frequency sea level oscillations were also recorded at Bakar (30 cm) in the northern Adriatic. It is also important to note the simultaneous intensification of high-frequency air pressure oscillations and high-frequency sea level oscillations (Figs. 5 and 6).

The presence of the fundamental Adriatic seiche (with a period of approximately 21.2 h) is also evident in the residual time series (Fig. 6, left, 1.5 periods seen). The seiche was likely triggered by the surge, as it emerged after the impact of the extratropical cyclone on the Adriatic sea level weakened, presenting a typical situation for the onset of the Adriatic seiche (Cerovečki et al. 1997; Raicich et al. 1999).

**Fig. 7:**
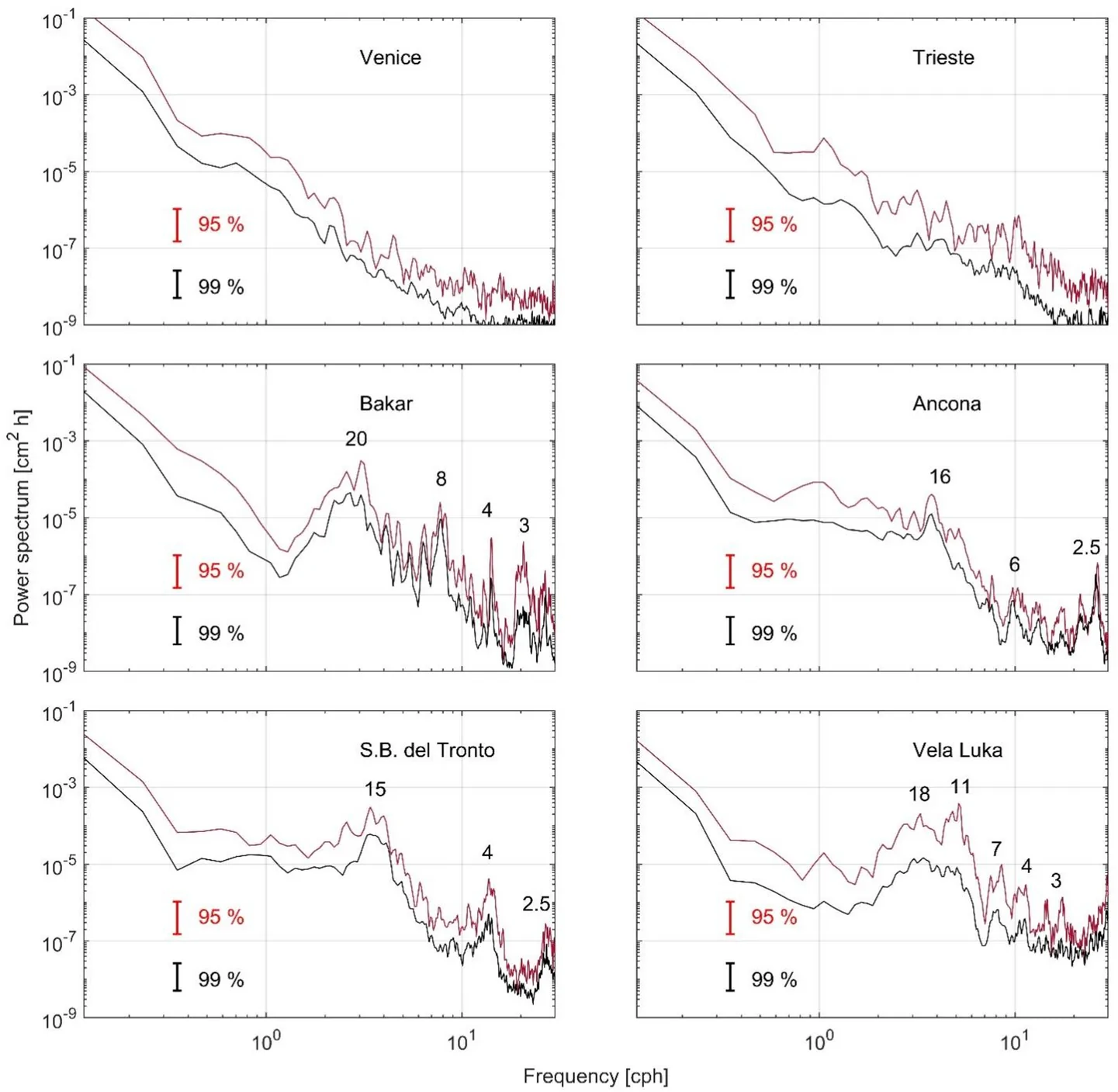
Power spectra of the residual sea level during the event (red line) and during the background period (black line). Spectra for Venice and Trieste stations are shown in the first row, for Bakar and Ancona stations in the second row, and for San Benedetto del Tronto and Vela Luka stations in the third row. Rows represent stations in which high-frequency sea level oscillation do not contribute significantly (first row), moderately contribute (second row) and strongly contribute (third row) to extremes of residual time series. The numbers indicate the periods (in minutes) of significant peaks

We also analysed the extreme event of 29–30 October 2018 in the frequency domain. Power spectra of sea level time series are presented in Fig. 7 for six selected tide gauge stations (Venice, Trieste, Bakar, Ancona, San Benedetto del Tronto and Vela Luka). A compound extreme was recorded at all these stations. The spectra were computed both for the duration of the event and for a background period characterised by only weak sea level oscillations (referred to as the background spectrum), following the methodology described in Sect.  2.1. Data collection and analysis.

As expected, at all stations, background spectra exhibit lower energy levels compared to the event spectra. An increase in spectral energy is observed during the 29–30 October event, both at longer periods associated with storm surge dynamics (*T* > 2 h), and at shorter periods corresponding to the intensification of shelf, bay and harbour seiches, i.e., with potential meteotsunamis. Differences in power spectra can be observed among stations from different parts of the Adriatic. For Trieste and Venice stations, no significant spectral peaks appear at shorter (*T* < 2 h) periods. For Bakar and Ancona stations, multiple significant peaks are present. In Bakar, peaks are observed around 20, 7, 4, and 3 min, while in Ancona, peaks are found at approximately 17, 6, and 3 min. For the last two stations, San Benedetto del Tronto and Vela Luka, a single dominant peak is present: a broad peak at around 11–18 min in Vela Luka and at 15 min in San Benedetto del Tronto. These periods correspond to the natural seiche periods of the bays and harbours in which these tide gauge stations are located (Šepić et al. 2016; Denamiel et al. 2018).

## Forecasting compound extremes

### Joint atmospheric and oceanographic modelling

#### Atmospheric modelling

Numerical modelling of atmospheric processes leading to meteorological tsunamis is extremely complex. For example, Belušić et al. (2007) examined a wide range of parameterization schemes within the MM5 atmospheric model in an effort to accurately reproduce the observed atmospheric pressure fields recorded during the middle Adriatic meteotsunami of 27 June 2003. A similar approach was followed by Šepić et al. (2009) and Horvath et al. (2018), who modelled the atmospheric component of the Adriatic meteotsunamis of 22 August 2007 and 25–26 June 2014, respectively. Despite multiple efforts to improve the atmospheric model, none of the mentioned research teams were able to fully reproduce the observed tsunamigenic atmospheric pressure disturbances. Horvath and Vilibić (2014) did a comprehensive numerical atmospheric modelling sensitivity study using the WRF model for simulation of the atmospheric conditions during Boothbay Harbor meteotsunami on 28 October 2008. For this particular event they showed that model, in reproducing the event, was more sensitive to the horizontal grid spacing, initial and boundary conditions, the choice of convective parameterization and simulation lead time and to a lesser extent to a choice of microphysics parameterization and the nesting strategy (one-way or two-way).

A different approach was tried out by authors who designed their models to be operational, i.e., by authors that use the same model setup regardless of the specific event. This approach has been applied, for example, by Denamiel et al. (2019a); Mourre et al. (2021) for forecasting the Adriatic and Balearic meteotsunamis, respectively. However, their models, due to the lack of event-specific model tuning, also often fail to reproduce observed atmospheric disturbances. Given that the goal of our study was to assess the potential for real-time meteotsunami forecasting, the latter approach was also adopted here, namely, a single simulation of atmospheric fields using the WRF model was performed without any additional model configuration changes and event specific fine-tuning.

**Fig. 8:**
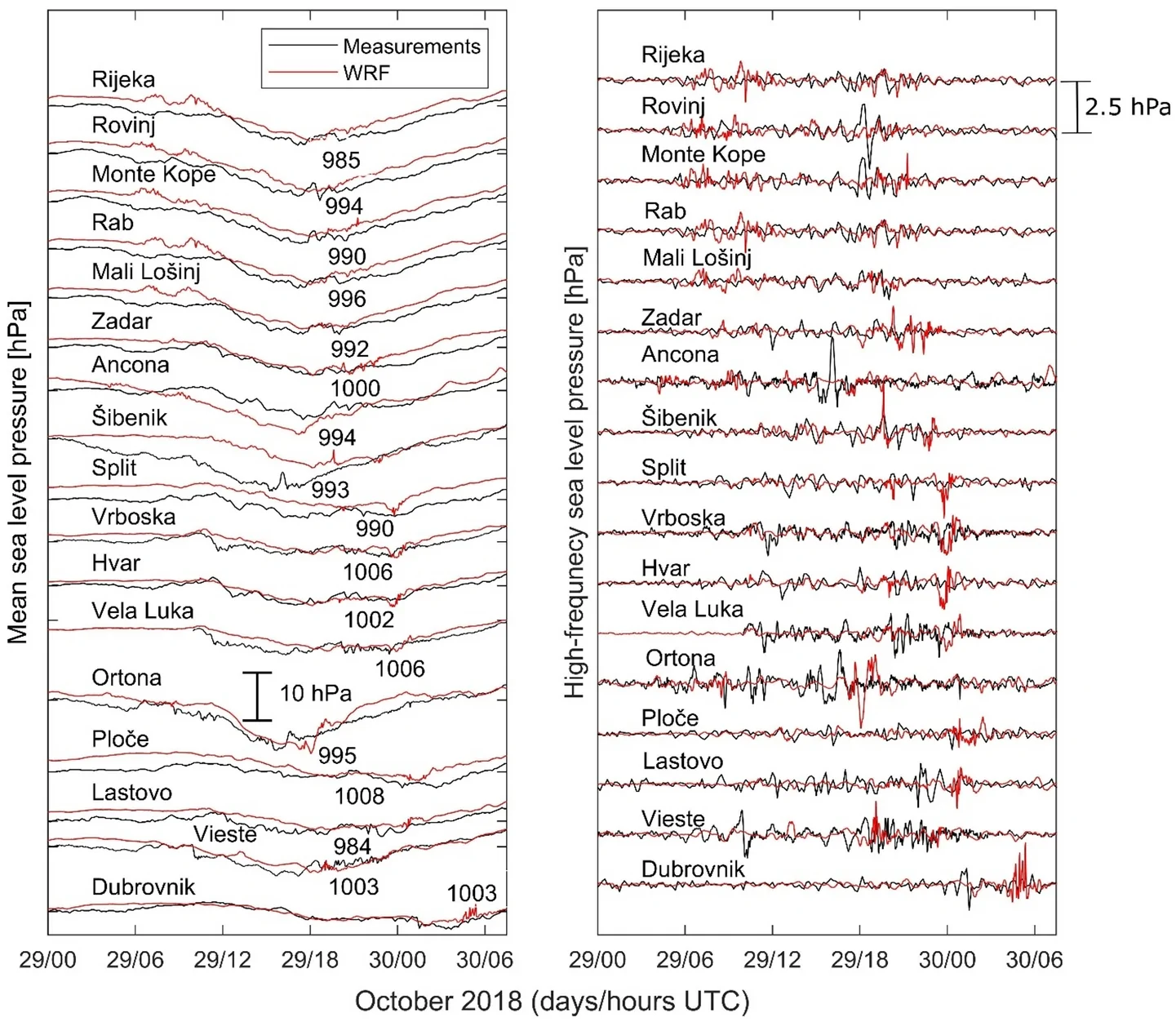
Left: mean sea level pressure time series; Right: high-frequency (*T* < 2 h) component of air pressure. Measured values are shown in black, and modelled values in red. The series are vertically offset by an arbitrary amount along the y-axis to enhance visual clarity

A comparison between measured and modelled original and high-frequency air pressure series is given in Fig. 8 with modelled series originating from the WRF model grid points closest to meteorological stations. The original air pressure time series (i.e., time series containing both low and high-frequency components) are satisfactorily reproduced (Fig. 8, left). The intensity of the pressure drop during cyclone passage and the timing of the pressure minimum roughly match between observations and the model. Regarding the high-frequency pressure oscillations, it is evident that these oscillations intensify at the appropriate time in the model across all stations. However, the oscillation periods and pressure change rates are less consistent with the observed data (Fig. 8, right).

Another measure of the model’s performance in reproducing air pressure series can be seen in Table 3, which shows the root mean square error (*RMSE*) and *Bias* obtained by comparing measured and modelled values of both the original air pressure series and the high-frequency components of air pressure series. Regarding the original air pressure series, the *RMSE* ranges from 1 to 1.48 hPa and *Bias* from − 1.05 to 0.51 hPa, indicating high model skill. Regarding the high-frequency component, relatively small *Bias-es* were calculated as well (Table 3). However, herein as both measured and modelled HF series oscillate around zero, *Bias* values should be taken with caution. Relatively higher *RMSE* values indicate that phase and exact timing of HF oscillations were not accurately reproduced.

**Table 3:** Root mean square error (*RMSE*) and *Bias* obtained by comparing measured and modelled original and high-frequency components of air pressure series

Station	RMSE air pressure [hPa]	Bias air pressure [hPa]	RMSE high-frequency component [hPa]	Bias high-frequency component [hPa]
Dubrovnik	0.71	−0.26	0.27	0.005
Vieste	1.12	−0.28	0.35	0.002
Lastovo	0.75	−0.03	0.33	−0.006
Ploče	1.15	−0.43	0.24	−0.005
Ortona	1.22	0.01	0.39	0.003
Vela Luka	1.19	−0.73	0.33	0.001
Hvar	0.75	0.12	0.29	−0.006
Vrboska	1.33	−1.05	0.3	0.001
Split	0.92	0.51	0.28	−0.006
Šibenik	1.05	−0.7	0.25	−0.004
Ancona	1.48	−0.75	0.37	0.009
Zadar	0.95	−0.31	0.25	0.006
Mali Lošinj	0.9	−0.35	0.23	−0.003
Rab	1.2	−0.81	0.29	0.004
Monte Kope	0.74	0.14	0.31	−0.003
Rovinj	1.17	−0.79	0.31	−0.003
Rijeka	0.82	0.06	0.29	0.004

**Fig. 9:**
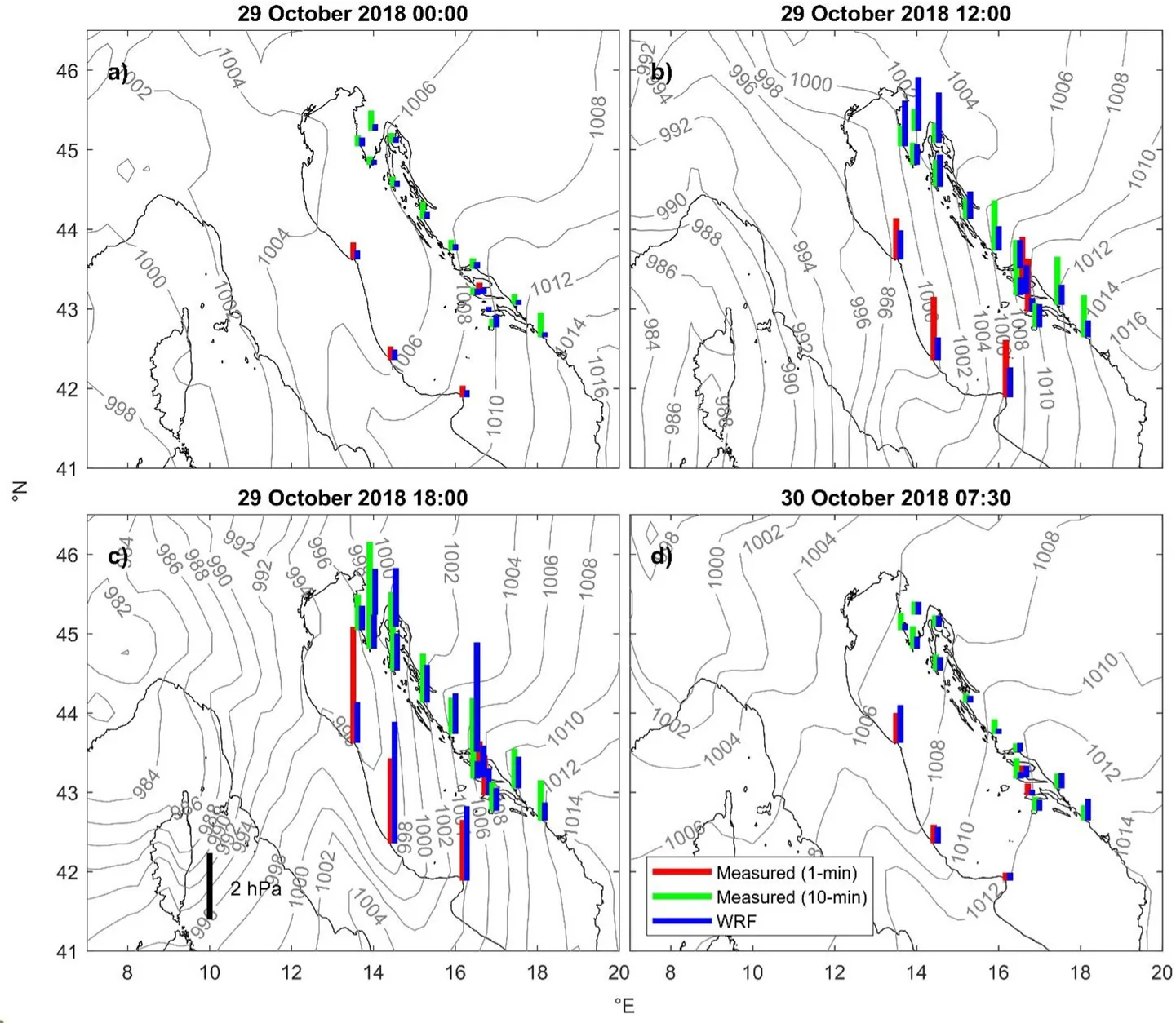
The range of high-frequency atmospheric pressure oscillations in the time frame of two hours before and after the indicated time, at: (a) 29 October 2018 (00:00 UTC), (b) 29 October 2018 (12:00 UTC), (c) 29 October 2018 (18:00 UTC) and (d) 29 October 2018 (07:30 UTC). Blue bars present modelled values while red and green bars indicate values obtained from measurements with 1-minute and 10-minute sampling rates respectively. Mean sea level pressure isobars, retrieved from the ERA5 reanalysis, are shown in grey at the times indicated in the figure (07:00 UTC on 30 October). The isobar values are expressed in hPa. Reanalysis data were used due to their superior spatial coverage and more visually appealing representation compared to that of the WRF model (domain d02)

A general agreement between WRF model and observations with respect to high-frequency pressure oscillations is further demonstrated in Fig. 9, which displays the ranges of measured and modelled high-frequency pressure oscillations at specific time intervals (two hours before and after the indicated moment). Around the first selected time point, 12:00 UTC on 29 October, no significant oscillations are observed in either measured or modelled pressure series (Fig. 9a). At this time, the extratropical cyclone was still located over the western Mediterranean, although moderate southeasterly winds were already blowing across the southern and central Adriatic.

As the cyclone approached the northern Adriatic by 12:00 UTC on 29 October (Fig. 9b), a tightening of isobars was observed over the Adriatic, accompanied by strengthening of southeasterly winds and a decrease in mean sea level pressure. The arrival of the cyclone coincided with an intensification of high-frequency air pressure oscillations, observable in both the measured and modelled data. Although the ranges of measured and modelled oscillations did not match perfectly across all stations, particularly along the western Adriatic coast, the model did reproduce the occurrence of pronounced high-frequency pressure oscillations at the time of extratropical cyclone approach.

The continued approach of the centre of the cyclone toward the northern Adriatic led to a further decrease in pressure and additional strengthening of southeasterly winds over the Adriatic (18:00 UTC, 29 October, Fig. 9c). At this time, a further intensification of high-frequency air pressure oscillations was observed in both the measured and modelled data across the Adriatic Sea. The largest range (maximum-minimum) of measured oscillations was recorded at Ancona (3.5 hPa), and the largest range of modelled oscillations at Split (3.3 hPa).

After the cyclone had passed, conditions over the Adriatic began to calm down. This calming was reflected in an increase in mean sea level pressure, a weakening of the winds at 10 m, and a reduction in the intensity of high-frequency pressure oscillations (07:30 UTC, 30 October, Fig. 9d).

Additionally, for the calculated ranges, *RMSE* and *Bias* values are presented in Table 4. From the original high-frequency time series, the ranges were computed as the difference between maximum and minimal values in 2 h windows. The presented values further demonstrate the agreement between WRF and measurements. Although the values of *RMSE* and *Bias* are higher than in Table 3, the difference arise from the fact that the values in Table 4 are computed from magnitudes of oscillations (difference between maximum and minimal values in a 2-h period) giving larger values in general. Maximum *RMSE* is obtained for Vela Luka (0.66) and the smallest for Ploče (0.29) while regarding the *Bias*, largest absolute values are obtained for Lastovo and Ancona (0.28) which also show largest values in Table 3. Smallest absolute values of *Bias* are obtained for Rovinj (0.0009) and Vrboska (0.007) which also has the smallest absolute *Bias* value in Table 3.

To conclude, the atmospheric model successfully reproduced the low-frequency component of air pressure variations. Regarding the high-frequency component, the model was able to reproduce the spatiotemporal distribution of these oscillations; however, their exact intensity, period, and timing were not represented with full accuracy.

**Table 4:** Root mean square error (*RMSE*) and *Bias* obtained by comparing ranges of measured and modelled high-frequency pressure oscillations

Station	RMSE high-frequency component [hPa]	Bias high-frequency component [hPa]
Dubrovnik	0.65	0.116
Vieste	0.44	−0.204
Lastovo	0.62	−0.282
Ploče	0.29	0.014
Ortona	0.63	−0.028
Vela Luka	0.66	−0.483
Hvar	0.49	0.067
Vrboska	0.51	0.007
Split	0.45	0.084
Šibenik	0.37	0.22
Ancona	0.56	−0.281
Zadar	0.52	0.211
Mali Lošinj	0.37	−0.062
Rab	0.48	0.013
Monte Kope	0.48	0.124
Rovinj	0.53	0.0009
Rijeka	0.53	0.259

#### Ocean modelling

**Fig. 10:**
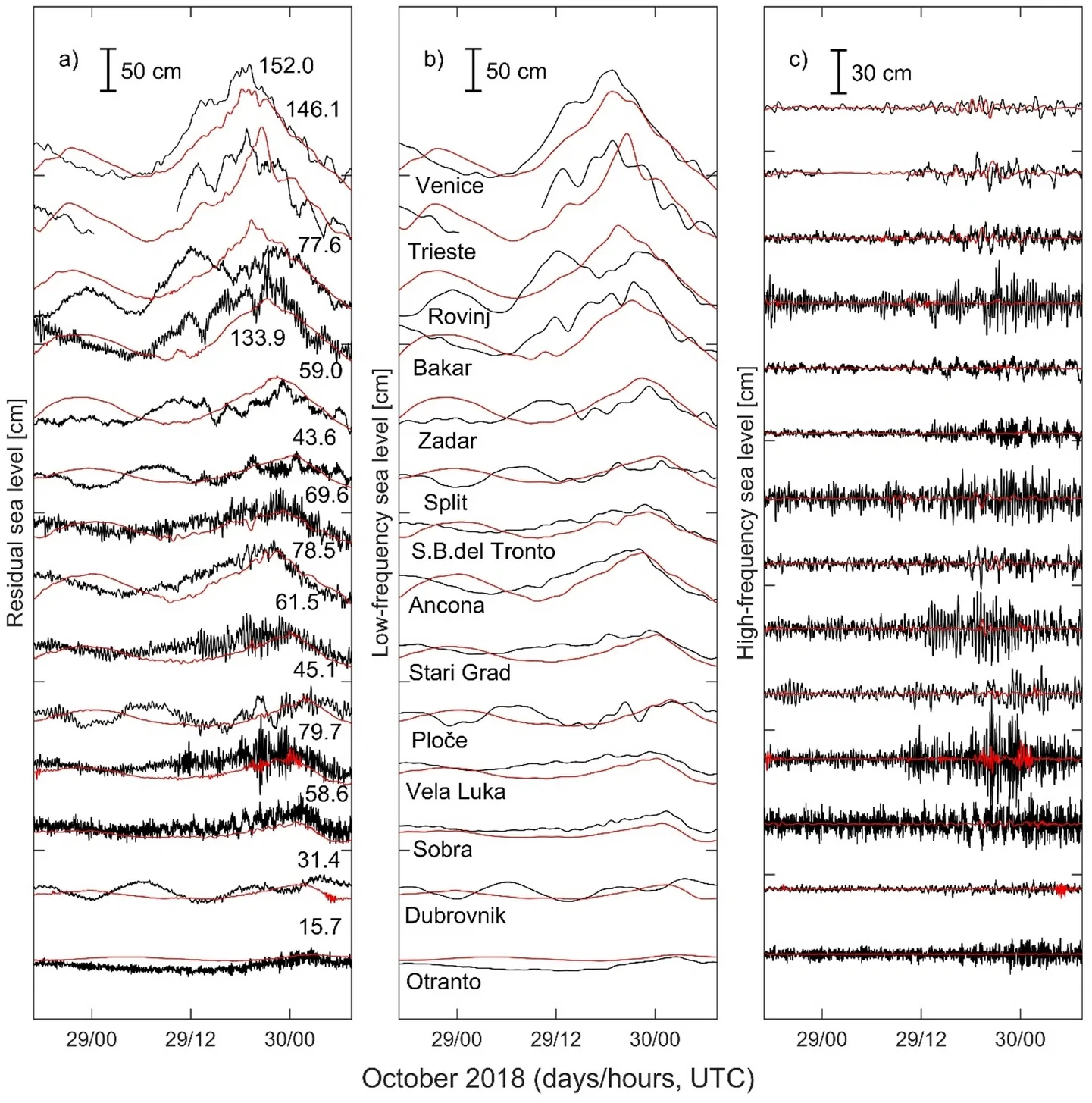
Observed (black) and modelled (red) sea level oscillations at locations of tide gauges during the compound extreme of 29–30 October 2018. Presented in (**a**) are the residual; (**b**) low-frequency; and (**c**) high-frequency time series. Maximum values of the measured residual time series are denoted in cm and presented in pane a). The time series were vertically offset by an arbitrary amount along the y-axis to enhance visual clarity

The oceanographic model SCHISM was forced using output fields from the WRF atmospheric model for the period from 12:00 UTC on 28 October 2018 to 07:30 UTC on 30 October 2018. The interpolation from WRF to SCHISM grid was necessary due to the unstructured grid used in SCHISM. A comparison of measured and modelled sea level time series is shown in Fig. 10, where Fig. 10a presents the comparison of the residual, Fig. 10b of the low-frequency and Fig. 10c of the high-frequency sea level time series. The model satisfactorily reproduces the shape, height, and timing of the storm surge at most stations, which is evident from Fig. 10a and b. The best agreement was achieved at Venice, Bakar and Ancona, while poorer agreement was achieved at Trieste (where the modelled maximum lags behind the observed one) and at Rovinj, where the observed maximum exhibits two peaks while the modelled time series have only one. The discrepancy in the onset time of the maximum which can be seen at some stations, such as Trieste, could be the result of the proximity of the location to the boundary of the atmospheric model which can result with uncertainties in the atmospheric forcing. Additionally, at all stations a discrepancy between the modelled and measured time series can be seen in the first hours, after the start of the forcing (after 12:00 on the 28 October). This is likely due to the model initialization, as the oceanographic model (SCHISM) did not have sufficient time to adjust to the WRF atmospheric forcing. In contrast to the reproduction of the storm surge (low-frequency sea level oscillations, Fig. 10b), the reproduction of high-frequency sea level oscillations is unsatisfactory (Fig. 10c). Statistical comparison of the modelled and measured values is given in Table 5 in which the *RMSE* and *Bias* for all stations, obtained by comparing, respectively, the residual, low-frequency, and high-frequency measured and modelled series are given. It can be observed that the *RMSE* of the residual series is highest precisely for the Trieste and Rovinj stations, exceeding 22.55 cm. Regarding the high-frequency component, it is most unsatisfactorily reproduced, that is, it has the highest *RMSE*, at the San Benedetto del Tronto, Stari Grad, and Vela Luka stations. For the last two mentioned stations, Stari Grad and Vela Luka, the intensification of modelled high-frequency values occurs at the same time as in the measurements, but due to the low modelled amplitudes, these stations have large *RMSE* values. We can confirm this by observing the *Bias*, which is among the lowest at these two stations, indicating that the measured and modelled series oscillate around zero in a similar way.

**Table 5:** Root mean square error (*RMSE*) and *Bias* obtained by comparing measured and modelled residual, low-frequency and high-frequency components of sea level

Station	Residual RMSE [cm]	Residual Bias [cm]	LF RMSE [cm]	LF Bias [cm]	HF RMSE [cm]	HF Bias [cm]
Otranto	10.65	9.35	10.12	9.33	3.23	0.013
Dubrovnik	10.1	−5.73	9.79	−5.72	1.78	−0.005
Sobra	11.63	−9.51	10.05	−9.5	5.61	−0.007
Vela Luka	15.37	−11.22	12.47	−11.22	8.88	−0.002
Ploče	11.56	−4.13	10.24	−4.1	4.34	−0.027
Stari Grad	13.41	−8.38	11.26	−8.39	7.09	0.01
Ancona	13.67	−3.14	12.79	−3.16	4.24	0.025
S. B. del Tronto	11.44	−4.91	8.95	−4.96	6.78	0.047
Split	11	−0.04	10.29	−0.06	3.2	0.026
Zadar	16.37	3.34	15.74	3.31	2.91	0.038
Bakar	21.29	−10.95	19.81	−10.97	6.3	0.021
Rovinj	22.55	6.93	21.83	6.92	3.64	0.011
Trieste	28.62	−9.63	26.7	−9.54	4.55	−0.08
Venice	19.48	−10.36	18.88	−10.38	3.26	0.013

**Fig. 11:**
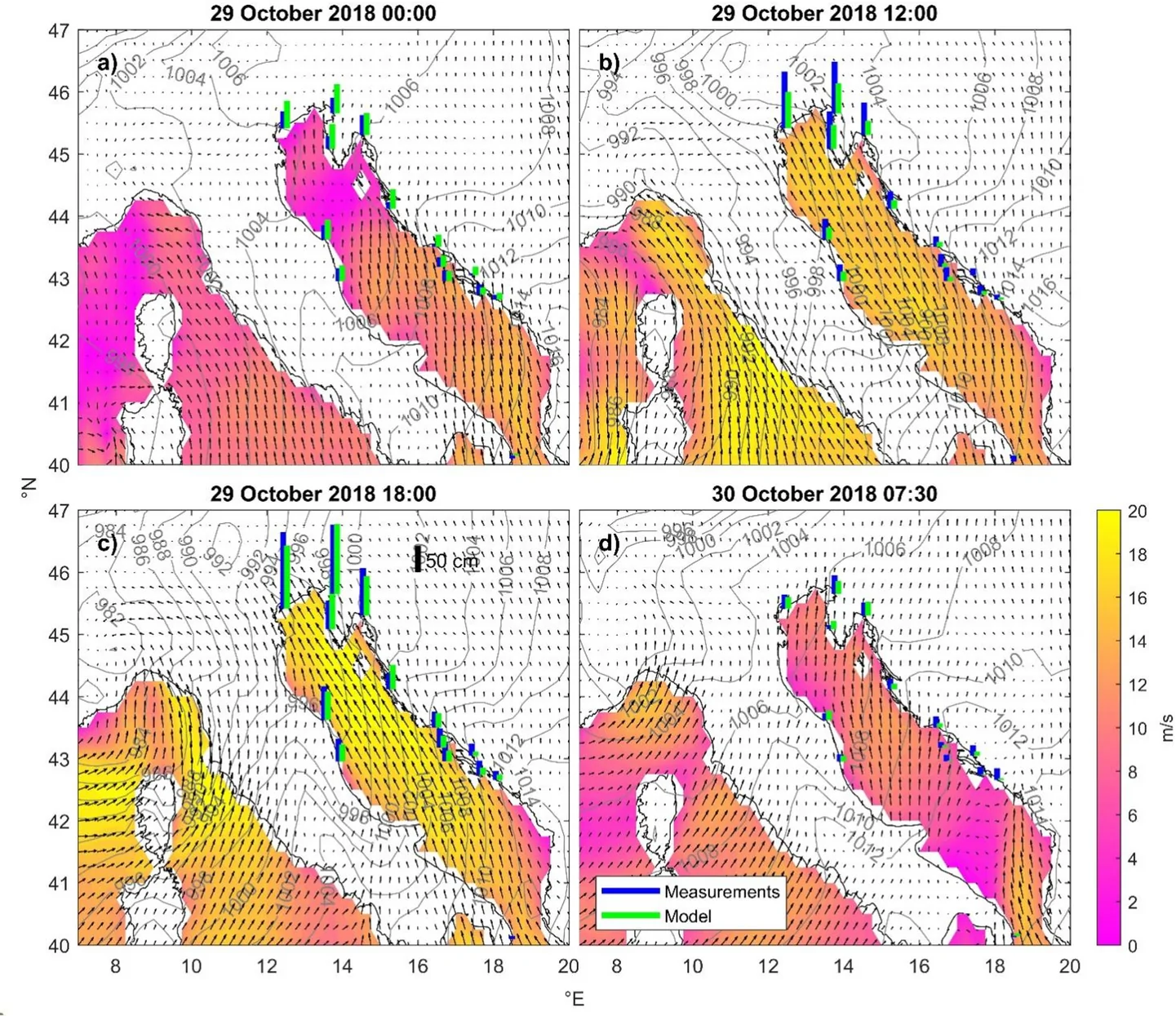
Maximum height of the low-frequency sea level component within a ± 2-hour window cantered on the indicated time point. Values are shown for tide gauge stations (blue bars) and for the SCHISM model closest points to the respective stations (green bars). Also presented are mean sea level pressure isobars from the ERA5 reanalysis (in hPa) (for 07:00 UTC, 30 October 2018). Wind directions (black arrows) and wind speeds at 10 m above the surface (shown only where wind speeds exceed 10 m/s) are also derived from the ERA5 reanalysis. Reanalysis data were used due to their superior spatial coverage and more visually appealing representation compared to that of the WRF model (domain d02)

Good agreement between the timing and magnitude of the low-frequency component of the measured and modelled time series is confirmed in Fig. 11, which presents the maximum values of the low-frequency component within a two-hour window before and after selected time points. Additionally, mean sea level pressure isobars, from the ERA5 reanalysis data, are shown for the specified time points (in grey). At the initial time step (00:00 UTC on 29 October), prior to the approach of the cyclone centre from the western Adriatic, the heights of the low-frequency sea level component were low (Fig. 11a). The subsequent approach of the extratropical cyclone toward the northern Adriatic, detectable at both 12:00 (Fig. 11b) and 18:00 UTC (Fig. 11c) on 29 October, was accompanied by the rise of the low-frequency sea level, with the highest recorded values observed at the northern Adriatic stations of Trieste (131.3 cm) and Venice (144.7 cm). This rise in sea level was caused by the decreased atmospheric pressure over the northern Adriatic (inverse barometric effect) and by the southeasterly wind, which, as previously described, resulted in the advection of seawater toward the northern Adriatic. Wind speeds were approximately 15 m/s at 12:00 and increased to around 20 m/s at 18:00 UTC across the entire Adriatic. A calming of the situation was observed at the final time point (07:30 UTC on 30 October 2018; Fig. 11d), when mean sea level pressure increased, the southeasterly wind weakened (with peak values up to 15 m/s over limited areas, while remaining below this threshold over most of the basin), and, accordingly, the heights of the low-frequency sea level component decreased.

**Fig. 12:**
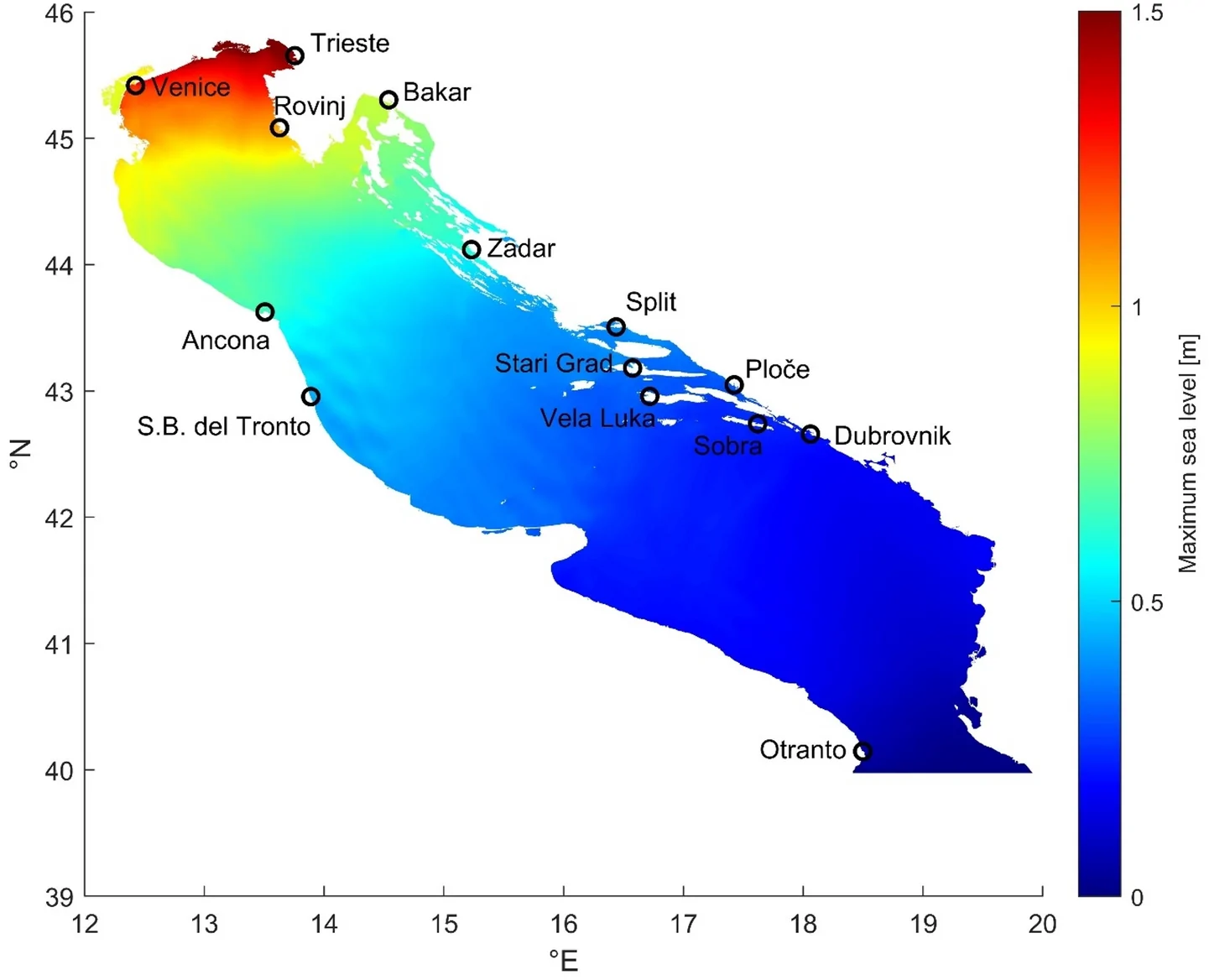
Spatial distribution of the maximum modelled residual sea level values during the entire simulation (28–30 October 2018). Locations, and names, of tide gauge stations are also given

Maximum values of the residual sea level are expected to occur at places where storm surge is the strongest, i.e., places where the mean sea level pressure was the lowest and wind fetch was the longest and where sea is shallowest – as wind effects is strongest over the shallowest sea (Eq. 1). For the compound extreme of 29–30 October 2018, the northern Adriatic is the place where both these effects have their maximum contribution, which is revealed by the measured and modelled sea level time series in Fig. 10 and the spatial distribution of the maximum modelled residual sea level values presented in Fig. 12.

We now turn our attention to the high-frequency sea level oscillations (Fig. 10c). It is evident from this figure that, as already stated, for the majority of stations (e.g., Bakar, Split, and San Benedetto del Tronto) the model fails to satisfactorily reproduce the high-frequency component. Although the model reproduces the timing of the intensification of high-frequency sea level oscillations, the amplitude of these oscillations remains significantly underestimated compared to observations. Nonetheless, at stations such as Venice, Trieste and Dubrovnik, the high-frequency component is reasonably well reproduced in terms of both timing and magnitude. It is important to note that at these stations the contribution of high-frequency oscillations to the total sea level is generally low. In contrast, for stations that have more relevant contribution of the high-frequency component to the residual signal, the model fails to capture the high-frequency sea level oscillations with sufficient accuracy. At all stations the intensity of modelled high frequency sea level oscillations remains too low. The highest modelled high-frequency oscillations were found at Vela Luka and Ancona: in Vela Luka, the observed maximum oscillation reached 46.9 cm, while the modelled value was 13.3 cm; in Ancona, the observed and modelled maxima were 15.0 cm and 5.4 cm, respectively.

#### Discerning the forecasting issues

To investigate the causes of mismatch between modelled and measured high-frequency sea level oscillations, further analyses were carried out, focusing again on the high-frequency component of atmospheric pressure (as this component is crucial for the generation of high-frequency ocean oscillations). While the atmospheric model generally captures the spatial and temporal characteristics of high-frequency air pressure oscillations (Fig. 9), discrepancies are evident in several key parameters, such as pressure change rates, oscillation periods and the propagation pathways of air pressure disturbances. This can be, in part, a consequence of the interpolating step that was used to get the WRF output onto the SCHISM grid but nonetheless needs to be more thoroughly investigated.

**Fig. 13:**
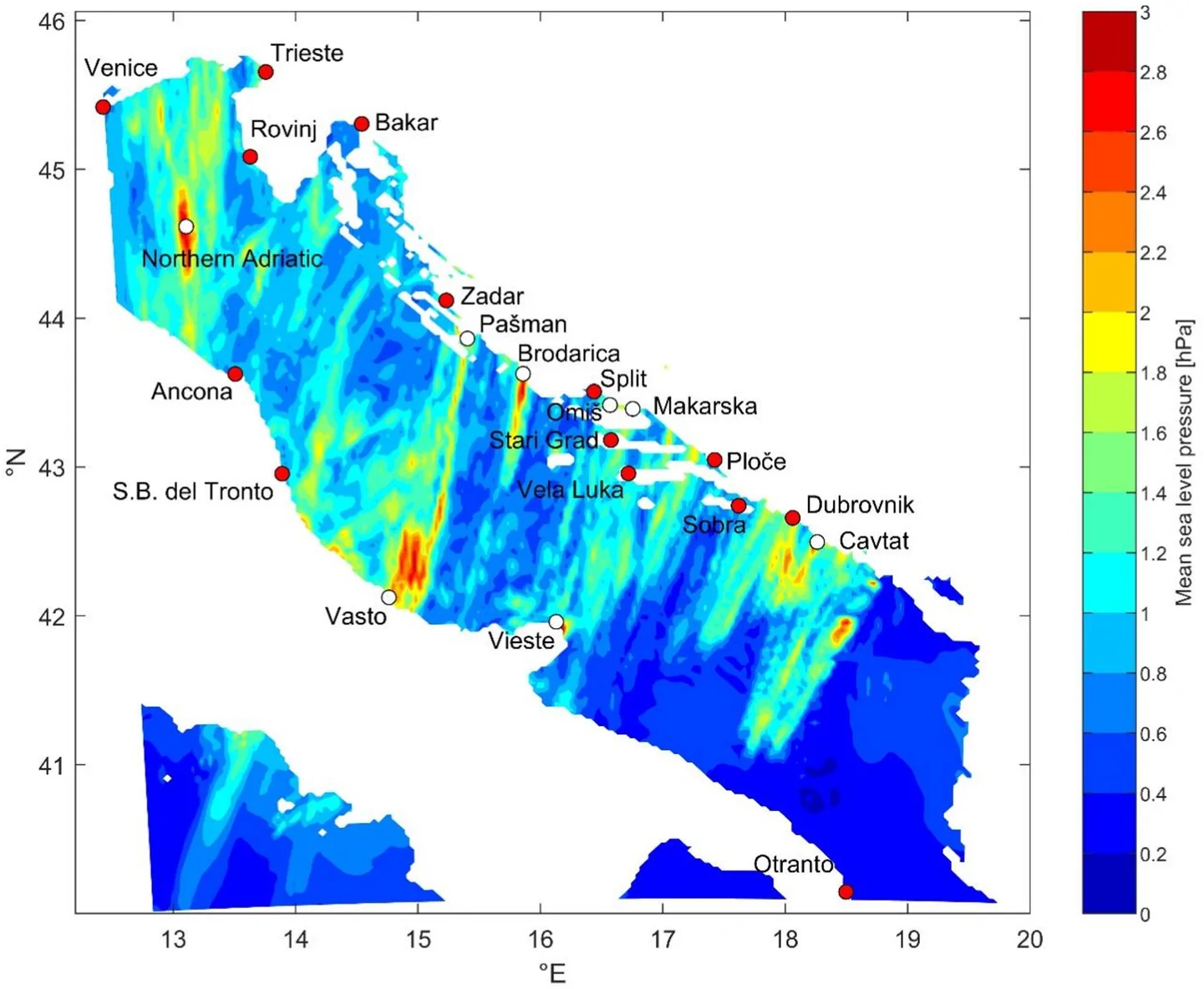
Maximum values of the high-frequency (*T* < 2 h) component of mean sea level pressure from the WRF model at SCHISM model points. Red circles denote the locations of tide gauges, while white circles indicate locations where large modelled values of high-frequency sea level oscillations are expected given the propagation path of modelled high-frequency atmospheric pressure disturbances (see Fig. 14)

The spatial distribution of the maximum values of modelled high-frequency air pressure oscillations is shown in Fig. 13, but only at points corresponding to SCHISM model points. The model reproduces pressure oscillations with heights of up to 3 hPa, comparable to measured heights. These oscillations are reproduced along narrow corridors oriented south-north in the northern Adriatic and southwest-northeast in the central and southern Adriatic. The paths are approximately 10–20 km wide, consistent with the findings of Šepić et al. (2016), who showed, by analysing air pressure series measured during 25–26 June 2014 meteotsunami Adriatic events, that cross-propagation pathways of tsunamigenic atmospheric disturbances in the Adriatic are precisely of these or slightly larger widths. From Fig. 13, it is possible to estimate the length over which modelled disturbances maintain full intensity, and it is up to approximately 50 km. For the event of 29–30 October 2018, the most intense air pressure oscillations were modelled over the open northern Adriatic, along the Vasto-Pašman transect, near Vieste, around Makarska and Omiš, and south of Dubrovnik (Fig. 13).

**Fig. 14:**
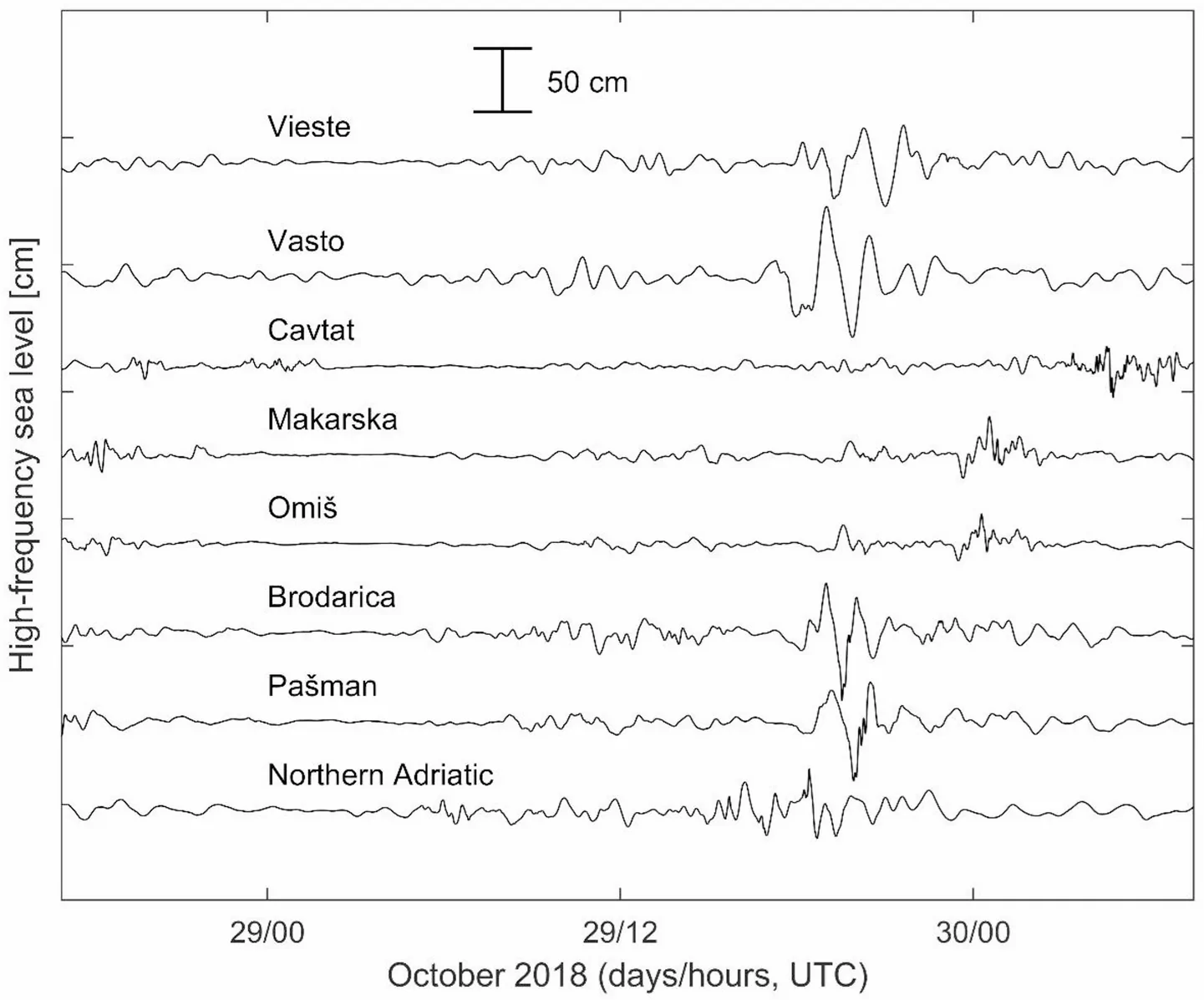
Modelled high-frequency sea level time series from locations denoted with white circles in Fig. 13. The time series were vertically offset by an arbitrary amount along the y-axis to enhance visual clarity

It is noteworthy that strong high-frequency air pressure oscillations were generally not reproduced over most tide gauge locations (marked as red circles in Fig. 13), nor along the paths leading to them. Given the strong high-frequency oscillations recorded at some of these tide gauges, it is likely that in reality, strong high-frequency pressure oscillations did pass over these stations or over paths leading to them. The assumption is supported by the fact that in the model, high-frequency sea level oscillations were indeed modelled at locations over which strong atmospheric pressure oscillations passed (white circles in Fig. 13 for atmospheric pressure; Fig. 14 for sea level oscillations). In several of these locations, such as Vasto, modelled high-frequency sea level oscillations exceeded 50 cm, matching the observed maximum in Vela Luka. In locations like Brodarica, where the modelled air pressure disturbances propagated directly toward the site, the high-frequency sea level response is highly amplified. These results support the conclusion that the ocean model, in addition to successfully reproducing the low-frequency sea level component, is also capable of simulating high-frequency sea level oscillations. However, exact reproduction of observed sea level oscillations is strongly dependent on ability of the atmospheric model to accurately reproduce timing, pathway, propagation velocity and rate of air pressure change of small-scale, high-frequency air pressure disturbances driving the ocean response.

### Combining real time air pressure measurements with ocean numerical modelling

Since joint use of atmospheric and ocean models, due to an unprecise reproduction of exact pathways of atmospheric pressure disturbances, did not give satisfactory results regarding the high-frequency sea level oscillations, we decided to force ocean numerical model with real-time air pressure measurements and focus on the output of the oceanographic model at Vela Luka as the primary target of the prediction system. Choice of Vela Luka as our focus location is supported by: (1) during 29–30 October 2018, a meteotsunami was observed and recorded at this location (Šepić and Orlić 2024) and (2) it is a well known meteotsunami hotspot (Orlić et al. 2010; Šepić and Orlić 2024). For this purpose, we conducted the following two experiments:

1. *EXPERIMENT VIESTE*: The numerical ocean model SCHISM was forced by the air pressure time series measured in Vieste (for locations see Fig. 1), under the assumption that the measured disturbance propagated without change in its properties across the Adriatic keeping constant speed and direction.
2. *EXPERIMENT VELA LUKA*: The numerical ocean model SCHISM was forced by the air pressure time series measured in Vela Luka, with the same assumptions as in the former (Vieste) case.

In both cases, the propagation velocity of atmospheric disturbance was determined as the mean values of wind velocity (over a narrow path from Italy to Croatia) at the 500-hPa level (from ERA5 reanalysis; Fig. 4), resulting in a speed of 30 m/s and a direction of 30° (~ north-northwest). The 500-hPa wind velocity was chosen as propagating velocity of atmospheric pressure disturbance in accordance with previous studies (Monserrat and Thorpe 1992, 1996; Šepić et al. 2015b), which demonstrated that propagation of tsunamigenic atmospheric disturbances is governed by mid-tropospheric winds. The resulting high-frequency sea level was than summed with the low-frequency sea level obtain from the earlier run of the SCHISM model, which was forced by the atmospheric WRF model data (Fig. 10b). By summing these two sea level time series we obtained the total sea level which was compared with the measured sea level to see whether this approach yields better results.

**Fig. 15:**
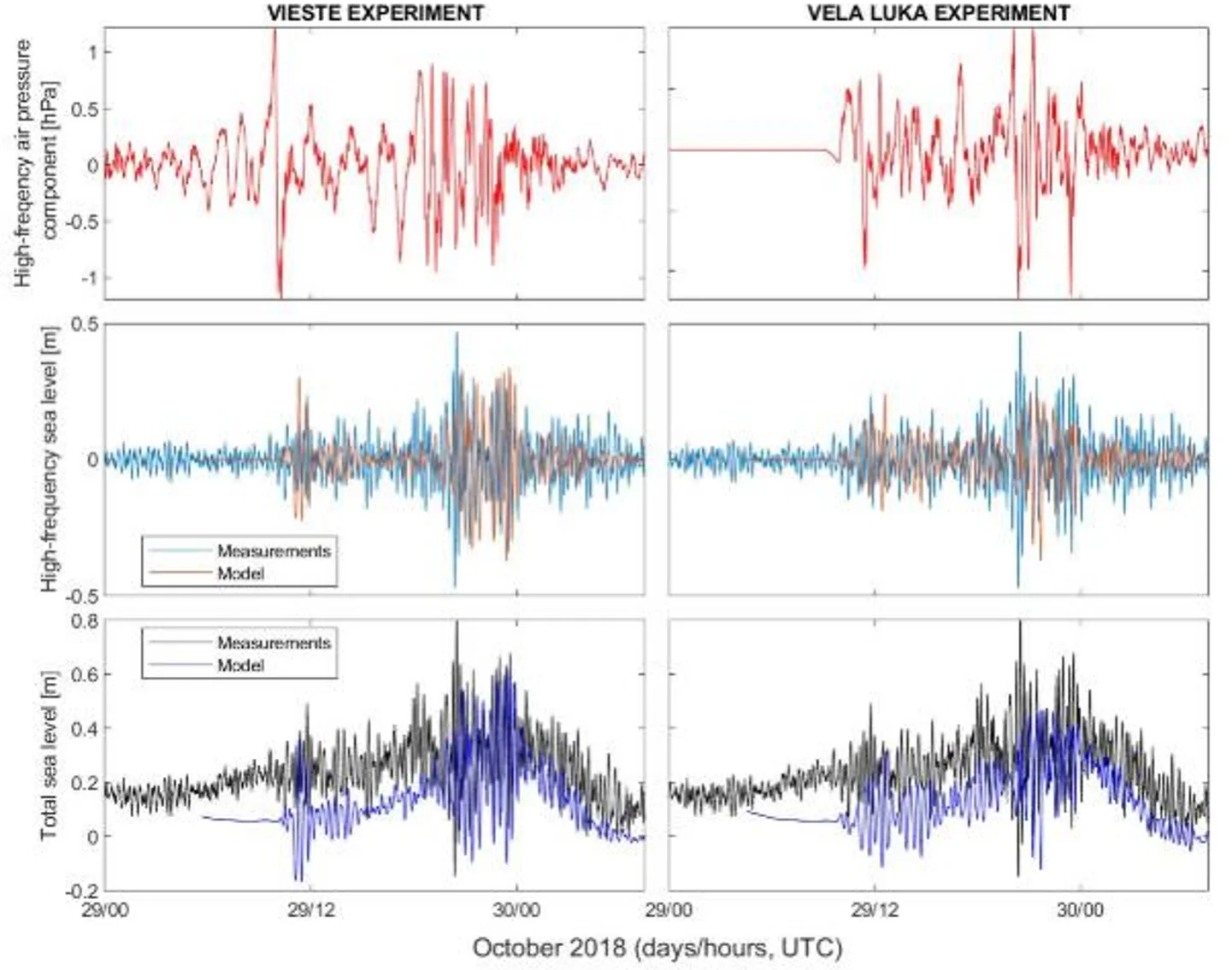
First row: measured high-frequency (*T* < 2 h) mean sea level pressure at Vieste (left) and Vela Luka (right). Second row: measured (blue) and modelled (red) high-frequency sea level at Vela Luka for VIESTE EXPERIMENT (left) and for VELA LUKA EXPERIMENT (right). Third row: total measured (black) and total modelled (blue) sea level at Vela Luka for VIESTE EXPERIMENT (left) and for VELA LUKA EXPERIMENT (right). Total modelled sea level is a sum of the low-frequency signal, obtained from the SCHIM model run forced by the WRF data, and the high-frequency signal derived from the SCHISM model run forced by the high-frequency part of measured air pressure series

Measured air pressure time series, as well as measured and modelled (for both experiments) high-frequency sea level time series and total sea level time series are shown in Fig. 15. It is evident that forcing the oceanographic numerical model with the measured air pressure time series results in much larger values of the high-frequency sea level oscillations relative to the ones obtained by forcing the model with the modelled values of the air pressure time series (Fig. 15 vs. Figure 10c). The maximum value of the high-frequency sea level oscillations in Vela Luka, for EXPERIMENT VIESTE (left side Fig. 15), was found to be 34 cm, which is more than 70% of the measured value (Table 6). For EXPERIMENT VELA LUKA, the maximum modelled high-frequency sea level in Vela Luka was 24 cm (Table 6). For both experiments Fig. 15 clearly shows intensification of the high-frequency sea level component at proper time. First at 12:00 UTC on the 29 October and again during the evening hours of the same day. It is notable that the timing of intensification of the modelled oscillations closely matches the timing of intensification of the observed oscillations.

In conclusion, much better results, regarding the reproduction of the high-frequency sea level oscillations, were obtained when the oceanographic numerical model was forced using the observed air pressure disturbances under the assumption that these air pressure disturbances propagated with the speed and direction of the 500 hPa wind. These values in combination with the low-frequency time series from the SCHISM model (Fig. 10b) give a much better agreement with the original sea level (last row in Fig. 15) than using only the SCHIM model for modelling the entire sea level (Fig. 10a). A disturbance propagating at the speed of 30 m/s, from the southwest coast of the Adriatic Sea towards the northeast, would require around 70 min to reach Vela Luka which allows sufficient time for issuing a warning. It is also indicative that better results were obtained when using air pressure time series measured at distanced stations (Vieste), than when using air pressure time series measured at the endangered location (Vela Luka) which implies that there is a strong potential for use of real time air pressure measurements originating from distanced stations for forecasting strong high-frequency sea level oscillations at the endangered location.

**Table 6:** For both runs (VIESTE and VELA LUKA) the table presents details of forcing used: maximum rate of change of the HF atm

Run type	Measured HF air pressure ocean model forcing	Maximum rate of change in the HF atm. pressure [hPa/min]	Speed [m/s] /direction of pressure disturbance propagation	Modelled maximum HF sea level [cm]	Measured maximum HF sea level [cm]	RMSE / Bias [cm]
EXPERIMENT VIESTE	Vieste	0.56	30 / 30°	34	47	12.88/ 0.01
EXPERIMENT VELA LUKA	Vela Luka	0.45	30 / 30°	24	47	11.47/ 0.016

pressure; propagation speed and direction of atmospheric disturbances; as well as maximum values of the modelled and measured sea level series at Vela Luka; and *RMSE* and *Bias*.

## Discussion and conclusions

Extreme values of sea level which were observed on the 29 and 30 October 2018 in the Adriatic were a consequence of processes acting on periods of both meteotsunamis (*T* < 2 h) and storm surges (*T* > 2 h). The synoptic situation during the event was such that it favoured the creation of both types of processes, as seen in Fig. 4 and described by earlier research (Vilibić and Šepić 2009; Lionello et al. 2012). Favourable synoptics for generation of long ocean waves over the Adriatic Sea, strongest of which are meteotsunamis, entail (i) relatively uniform MSLP field over the Adriatic Sea with low surface air pressure to the west of the Adriatic; (ii) inflow of warm and dry air from the southwest at 850 hPa into the Adriatic; (iii) presence of a strong southwesterly jet stream at 500 hPa and (iv) unstable atmospheric layers (Šepić et al. 2015c). Although this is the most common synoptic state during which meteotsunamis in the Adriatic occur is it not the only one. Intense long ocean waves and meteotsunamis can also happen in the presence of a cyclone (Ruić et al. 2025) which is the main driver of the storm surge (Lionello et al. 2012). However, conditions (ii)–(iv) are mostly kept in those situations as well.

An extreme event, in which both processes occur simultaneously, is called a compound extreme, and is hard to predict because of wide range of temporal and spatial scales at which it occurs.

Because the storm surge occurs due to atmospheric processes occurring on synoptic spatial scales (> 100 km) which are usually well reproduced by atmospheric numerical models, forecast of storm surges is usually satisfactory. This is also highlighted in the presented study, mainly in Figs. 10, 11 and 12. On the other hand, since meteotsunamis are smaller scale processes generated by atmospheric disturbances of limited spatial (10–100 km) and temporal (10s of minutes) dimensions they tend to be harder to predict. For a destructive meteotsunami to develop, multiple resonant processes are required to act jointly. Specifically, an atmospheric pressure disturbance characterized by a high rate of pressure change (several hPa over a few minutes) must propagate over the sea with an appropriate direction and speed to allow resonant processes to occur, e.g., Proudman resonance, (Proudman 1929) or Greenspan resonance (Greenspan 1956), and must be further amplified by shoaling effect or harbour resonance (Raichlen 1966) or both. As demonstrated by numerous authors (Belušić et al. 2007; Šepić et al. 2009; Orlić et al. 2010; Horvath et al. 2018; Denamiel et al. 2019a), as well as by the present study, it is extremely difficult to reproduce accurately such smaller scale atmospheric disturbances using atmospheric models. Consequently, the joint one- or two-way coupled numerical atmospheric-ocean models often fail to reproduce the magnitude of high-frequency sea level oscillations at observation stations. Nonetheless, a few systems have been created for meteotsunamis prediction, such as the Adriatic system ADCIRC described by Denamiel et al. (2019a, 2019b) (non-operational at the moment) and the Balearic Islands system, which is based on synoptic forecast since 1985 (Jansà and Ramis 2021), but nowadays supplemented by high-resolution numerical ocean modelling (Mourre et al. 2021; Romero 2024).

Results of the oceanographic model strongly depend on the forcing data, as shown in our study. The atmospheric WRF model successfully reproduced low-frequency component of atmospheric signal, i.e., an extratropical cyclone which propagated from the western Mediterranean towards the Adriatic and created strong pressure gradients and Sirocco winds over the Adriatic Sea. As for the higher-frequency oscillations, WRF model performed well on general statistical level, modelled high-frequency air pressure oscillations intensified at proper intervals reaching values similar to the measured ones (Fig. 9). However, the precise details of atmospheric pressure disturbances were not reproduced well: there was a mismatch between modelled and measured trajectories, speeds and directions, intensities, and durations of the atmospheric disturbances at individual locations. It is clearly evident that the pressure disturbances in the atmospheric model were of comparable intensity to the observed ones (Fig. 8), but since their paths were narrow (Fig. 13), this resulted in a sufficiently strong disturbance slightly deviating from the “ideal” (recorded) path, thus preventing the oceanographic model from producing an intensification of the high-frequency sea level component at the location at which this intensification was measured. This was one of the reasons for which we adopted the strategy of choosing one location, namely Vela Luka, as the primary target for the prediction of the hybrid approach in which the oceanographic model is forced by the air pressure measurements. We forced SCHISM with measured air pressure time series (measured at Vieste and Vela Luka, Sect.  4.2. and Fig. 15), under the assumption that these disturbances do not change shape and that they propagate with speed and direction corresponding to the wind at the 500-hPa level. Using measured air pressure time series resulted with significantly better reproduction of high-frequency sea level oscillations in Vela Luka, implying that, at this time, it is better to use real time air pressure measurements than high-resolution atmospheric models to forecast meteotsunamis.

To conclude, the steps of a potential forecasting system for compound extremes can be defined as follows:

1. High-resolution (1–4 km) modelling of atmospheric variables (10 m wind speed and mean sea level pressure) with minute-level output.
2. Forcing of an oceanographic model (e.g., SCHISM) with atmospheric model outputs (10-m wind speed and mean sea level pressure) for the purpose of storm surge reproduction.
3. Real-time forcing of the oceanographic model with air pressure measurements from a station located sufficiently far from the potentially affected site, in order to forecast high-frequency sea level oscillations (and potential meteotsunamis).
4. Combining the low-frequency sea level, obtained from (2) with the high-frequency sea level from (3) to get the total forecasted sea level.

Specifically, for the observation site of Vela Luka, locations along the eastern Italian coast (e.g., Bari, Vieste), which lie along the direct path between the forecasted wind direction at the 500-hPa level and the threatened location, should be monitored. For propagating speed of atmospheric disturbances ranging from 20 to 50 m/s, the potential lead time for issuing a warning ranges from 105 to 42 min, as this is the estimated time it takes for a disturbance to travel from the Italian coast to Vela Luka. Meteotsunami-focused simulations (step 3) should be repeated every ~ 10 min to maximize forecast accuracy. Naturally, this presents a challenge: the runtime of each simulation must not exceed 10 min. Such a system (steps 1 through 4) has also the potential to more successfully reproduce compound extremes, as clearly suggested by the results presented in this analysis.

The strength of this prediction method lies in the combination of two main factors. First, the numerical modelling (step (2)) provides a stable and reliable way of reproducing the storm surge component as demonstrated herein. Second, the use of real-time atmospheric forcing (step (3)) enables the high-frequency sea level component at selected location to be reproduced with good accuracy. Together, these factors provide a robust foundation for future development of the prediction system. One possible enhancement would be to incorporate additional atmospheric pressure stations, allowing predictions of high-frequency sea level oscillations to be generated for other locations of interest. One of the challenges for this enhancement is to find the adequate pairing of air pressure stations with sea level locations at which predictions are made. The stations need to be sufficiently distant from the prediction locations to provide enough time for possible warnings to be issued, while not that far apart so that the air pressure disturbances changes drastically while traveling. If there are multiple pairs of stations which are satisfactory, a development of a semi-automated approach could be made in which measured air pressure values from various stations could be interpolated over the whole basin to give basin-wide sea level predictions.

## References

[CR1] Anzidei M, Alberti T, Vecchio A, Loizidou X, Orthodoxou D, Serpelloni E, Falciano A, Ferrari C (2024) Sea level rise and extreme events along the Mediterranean coasts: the case of Venice and the awareness of local population, stakeholders and policy makers. Rend Fis Acc Lincei 35:359–370. 10.1007/s12210-024-01236-x

[CR2] Belušić D, Strelec Mahović N (2009) Detecting and following atmospheric disturbances with a potential to generate meteotsunamis in the Adriatic Sea. Phys Chem Earth. 10.1016/j.pce.2009.08.009

[CR3] Belušić D, Grisogono B, Klaić ZB (2007) Atmospheric origin of the devastating coupled air-sea event in the east Adriatic. J Geophys Res Atmos. 10.1029/2006JD008204

[CR4] Bertin X, Bakker A, van Dongeren A, Coco G, Andre G, Ardhuin F, Bonneton P, Bouchette F, Castelle B, Crawford W, Davidson M, Deen M, Dodet G, Guérin T, Inch K, Leckler F, McCall R, Muller H, Olabarrieta M, Tissier M (2018) Infragravity waves: from driving mechanisms to impacts. Earth Sci Rev. 10.1016/j.earscirev.2018.01.002

[CR6] Cavaleri L, Bajo M, Barbariol F et al (2019) The October 29, 2018 storm in Northern Italy – An exceptional event and its modeling. Prog Oceanogr 178:102178. 10.1016/j.pocean.2019.102178

[CR7] Cerovečki I, Orlić M, Hendershott MC (1997) Adriatic seiche decay and energy loss to the Mediterranean. Deep Sea Res Part I 44:2007–2029. 10.1016/S0967-0637(97)00056-3

[CR8] Chen F, Kusaka H, Bornstein R, Ching J, Grimmond CSB, Grossman-Clarke S, Loridan T, Manning KW, Martilli A, Miao S, Sailor D, Salamanca FP, Taha H, Tewari M, Wang X, Wyszogrodzki AA, Zhang C (2011) The integrated WRF/urban modelling system: development, evaluation, and applications to urban environmental problems. Int J Climatol 31:273–288. 10.1002/joc.2158

[CR5] Copernicus Climate Change Service, Climate Data Store (2023) ERA5 hourly data on single levels from 1940 to present. Copernicus Clim Change Service (C3S) Clim Data Store (CDS). 10.24381/cds.adbb2d47

[CR9] Denamiel C, Šepić J, Vilibić I (2018) Impact of Geomorphological Changes to Harbor Resonance During Meteotsunamis: The Vela Luka Bay Test Case. Pure Appl Geophys 175:3839–3859. 10.1007/s00024-018-1862-5

[CR10] Denamiel C, Šepić J, Ivanković D, Vilibić I (2019a) The Adriatic Sea and Coast modelling suite: Evaluation of the meteotsunami forecast component. Ocean Model 135:71–93. 10.1016/j.ocemod.2019.02.003

[CR11] Denamiel C, Šepić J, Huan X, Bolzer C, Vilibić I (2019b) Stochastic Surrogate Model for Meteotsunami Early Warning System in the Eastern Adriatic Sea. JGR Oceans 124:8485–8499. 10.1029/2019JC015574

[CR12] Ferrarin C, Bajo M, Benetazzo A, Cavaleri L, Chiggiato J, Davison S, Davolio S, Lionello P, Orlić M, Umgiesser G (2021) Local and large-scale controls of the exceptional Venice floods of November 2019. Prog Oceanogr 197:102628. 10.1016/j.pocean.2021.102628

[CR13] Greenspan HP (1956) The generation of edge waves by moving pressure distributions. J Fluid Mech 1:574–592. 10.1017/S002211205600038X

[CR14] Heidarzadeh M, Rabinovich AB (2021) Combined hazard of typhoon-generated meteorological tsunamis and storm surges along the coast of Japan. Nat Hazards 106:1639–1672. 10.1007/s11069-020-04448-0

[CR15] Hersbach H, Bell B, Berrisford P, Biavati G, Horányi A, Muñoz Sabater J, Nicolas J, Peubey C, Radu R, Rozum I, Schepers D, Simmons A, Soci C, Dee D, Thépaut J-N (2023) ERA5 hourly data on single levels from 1940 to present. Copernicus Climate Change Service (C3S) Climate Data Store (CDS). 10.24381/cds.adbb2d47

[CR16] Hong S-Y, Lim J (2006) The WRF Single-Moment 6-Class Microphysics Scheme (WSM6). Asia-Pac J Atmos Sci 42:129–151

[CR17] Horvath K, Vilibić I (2014) Atmospheric mesoscale conditions during the Boothbay meteotsunami: a numerical sensitivity study using a high-resolution mesoscale model. Nat Hazards 74:55–74. 10.1007/s11069-014-1055-1

[CR18] Horvath K, Šepić J, Prtenjak MT (2018) Atmospheric Forcing Conducive for the Adriatic 25 June 2014 Meteotsunami Event. Pure Appl Geophys 175:3817–3837. 10.1007/s00024-018-1902-1

[CR19] Jansà A, Ramis C (2021) The Balearic rissaga: From pioneering research to present-day knowledge. Nat Hazards 106(2):1269–1297.

[CR20] Kain JS (2004) The Kain–Fritsch Convective Parameterization: An Update. J Appl Meteor 43:170–181. 10.1175/1520-0450(2004)043%253C0170:TKCPAU%253E2.0.CO;2

[CR21] Kim J, Omira R (2024) Combined surge-meteotsunami dynamics: A numerical model for hurricane Leslie on the coast of Portugal. Ocean Modell 189:102368. 10.1016/j.ocemod.2024.102368

[CR22] Lionello P, Cavaleri L, Nissen KM, Pino C, Raicich F, Ulbrich U (2012) Severe marine storms in the Northern Adriatic: Characteristics and trends. Phys Chem Earth 40–41:93–105. 10.1016/j.pce.2010.10.002

[CR23] Medvedev IP, Vilibić I, Rabinovich AB (2020) Tidal resonance in the Adriatic Sea: observational evidence. JGR Oceans. 10.1029/2020JC016168

[CR24] Medvedev IP, Rabinovich AB, Šepić J (2022) Destructive coastal sea level oscillations generated by Typhoon Maysak in the Sea of Japan in September 2020. Sci Rep 12:8463. 10.1038/s41598-022-12189-2 PMC9120019 35589786

[CR25] Mlawer EJ, Taubman SJ, Brown PD, Iacono MJ, Clough SA (1997) Radiative transfer for inhomogeneous atmospheres: RRTM, a validated correlated-k model for the longwave. J Geophys Res 102:16663–16682. 10.1029/97JD00237

[CR26] Monserrat S, Thorpe AJ (1992) Gravity-Wave Observations Using an Array of Microbarographs In the Balearic Islands. Q J R Meteorol Soc 118:259–282. 10.1002/qj.49711850405

[CR27] Monserrat S, Thorpe AJ (1996) Use of Ducting Theory in an Observed Case of Gravity Waves. J Atmos Sci 53:1724–1736. 10.1175/1520-0469(1996)053<1724:UODTIA>2.0.CO;2

[CR28] Monserrat S, Vilibić I, Rabinovich AB (2006) Meteotsunamis: atmospherically induced destructive ocean waves in the tsunami frequency band. Nat Hazards Earth Syst Sci 6:1035–1051. 10.5194/nhess-6-1035-2006

[CR29] Morucci S, Coraci E, Crosato F, Ferla M (2020) Extreme events in Venice and in the North Adriatic Sea: 28–29 October 2018. Rend Fis Acc Lincei 31:113–122. 10.1007/s12210-020-00882-1

[CR30] Mourre B, Santana A, Buils A, Gautreau L, Ličer M, Jansà A, Casas B, Amengual B, Tintoré J (2021) On the potential of ensemble forecasting for the prediction of meteotsunamis in the Balearic Islands: sensitivity to atmospheric model parameterizations. Nat Hazards 106:1315–1336. 10.1007/s11069-020-03908-x

[CR31] Nakanishi M, Niino H (2006) An Improved Mellor–Yamada Level-3 Model: Its Numerical Stability and Application to a Regional Prediction of Advection Fog. Boundary-Layer Meteorol 119:397–407. 10.1007/s10546-005-9030-8

[CR32] Needham HF, Keim BD, Sathiaraj D (2015) A review of tropical cyclone-generated storm surges: Global data sources, observations, and impacts. Rev Geophys 53:545–591. 10.1002/2014RG000477

[CR33] Niu G-Y, Yang Z-L, Mitchell KE, Chen F, Ek MB, Barlage M, Kumar A, Manning K, Niyogi D, Rosero E, Tewari M, Xia Y (2011) The community Noah land surface model with multiparameterization options (Noah-MP): 1. Model description and evaluation with local-scale measurements. J Geophys Res 116:D12109. 10.1029/2010JD015139

[CR35] Orlić M, Pasarić M (2024) How to disentangle sea-level rise and a number of other processes influencing coastal floods? Rend Fis Acc Lincei 35:371–380. 10.1007/s12210-024-01242-z

[CR34] Orlić M, Belušić D, Janeković I, Pasarić M (2010) Fresh evidence relating the great Adriatic surge of 21 June 1978 to mesoscale atmospheric forcing. J Geophys Res 115:2009JC005777. 10.1029/2009JC005777

[CR36] Pasarić M, Orlić M (2001) Long-term meteorological preconditioning of the North Adriatic coastal floods. Cont Shelf Res 21:263–278. 10.1016/S0278-4343(00)00078-9

[CR37] Pattiaratchi CB, Wijeratne EMS (2015) Are meteotsunamis an underrated hazard? Phil Trans R Soc A. 10.1098/rsta.2014.0377 PMC4608035 26392619

[CR38] Pawlowicz R, Beardsley B, Lentz S (2002) Classical tidal harmonic analysis including error estimates in MATLAB using T_TIDE. Comput Geosci 28:929–937. 10.1016/S0098-3004(02)00013-4

[CR39] Pérez-Gómez B, García-León M, García-Valdecasas J, Clementi E, Mösso Aranda C, Pérez-Rubio S, Masina S, Coppini G, Molina-Sánchez R, Muñoz-Cubillo A, García Fletcher A, Sánchez González JF, Sánchez-Arcilla A, Álvarez Fanjul E (2021) Understanding Sea Level Processes During Western Mediterranean Storm Gloria. Front Mar Sci 8:647437. 10.3389/fmars.2021.647437

[CR40] Proudman J (1929) The Effects on the Sea of Changes in Atmospheric Pressure. Geophys J Int 2:197–209. 10.1111/j.1365-246X.1929.tb05408.x

[CR41] Pugh D, Woodworth P (2014) Sea-level science: understanding tides, surges, tsunamis and mean sea-level changes. Cambridge University Press, Cambridge; New York

[CR42] Rabinovich AB (2020) Twenty-Seven Years of Progress in the Science of Meteorological Tsunamis Following the 1992 Daytona Beach Event. Pure Appl Geophys 177:1193–1230. 10.1007/s00024-019-02349-3

[CR43] Rabinovich AB, Šepić J, Thomson RE (2023) Strength in Numbers: The Tail End of Typhoon Songda Combines with Local Cyclones to Generate Extreme Sea Level Oscillations on the British Columbia and Washington Coasts during Mid-October 2016. J Phys Oceanogr 53:131–155. 10.1175/JPO-D-22-0096.1

[CR44] Raichlen F (1966) Harbor resonance. Estuaryand Coastline Hydrodynamics. McGraw Hill Book Company, New York, pp 281–340

[CR45] Raicich F, Orlić M, Vilibić I, Malačič V (1999) A case study of the Adriatic seiches (December 1997). Nuovo Cimento Soc Ital Fis C 22:715–726

[CR46] Ramis C, Jansà A (1983) Condiciones meteorológicas simultáneas a la aparición de oscilaciones del nivel del mar de 640 amplitud extraordinaria en el Mediterráneo. Occidental Rev Geofisc 39:35–42

[CR47] Ramos-Alcántara J, Agulles M, Gomis D, Jordà G (2025) Quantifying the contributors to extreme sea level events in a Mediterranean microtidal region at high spatio-temporal resolution. Clim Dyn 63:167. 10.1007/s00382-025-07653-4

[CR48] Robinson AR, Tomasin A, Artegiani A (1973) Flooding of Venice: Phenomenology and prediction of the Adriatic storm surge. Q J R Meteorolog Soc 99:688–692. 10.1002/qj.49709942210

[CR49] Romero R (2024) TRAM: A new non-hydrostatic fully compressible numerical model suited for all kinds of regional atmospheric predictions. Q J R Meteorolog Soc 150(759):1124–1156. 10.1002/qj.4639

[CR50] Ruić K, Šepić J, Mlinar M, Međugorac I (2023) Contribution of high-frequency (T < 2 h) sea level oscillations to the Adriatic sea level maxima. Nat Hazards 116:3747–3777. 10.1007/s11069-023-05834-0

[CR51] Ruić K, Šepić J, Vojković M (2025) Synoptic patterns associated with high-frequency sea level extremes in the Adriatic Sea. Ocean Sci 21:1183–1203. 10.5194/os-21-1183-2025

[CR52] Rus M, Fettich A, Kristan M, Ličer M (2023) HIDRA2: deep-learning ensemble sea level and storm tide forecasting in the presence of seiches – the case of the northern Adriatic. Geosci Model Dev 16:271–288. 10.5194/gmd-16-271-2023

[CR53] Rus M, Mihanović H, Ličer M, Kristan M (2025) HIDRA3: a deep-learning model for multipoint ensemble sea level forecasting in the presence of tide gauge sensor failures. Geosci Model Dev 18:605–620. 10.5194/gmd-18-605-2025

[CR57] Šepić J, Orlić M (2024) Adriatic Meteotsunami Catalogue. Last access: 21 April 2024. https://projekti.pmfst.unist.hr/floods/meteotsunamis/

[CR58] Šepić J, Vilibić I, Belušić D (2009) Source of the 2007 Ist meteotsunami (Adriatic Sea). J Geophys Res 114:2008JC005092. 10.1029/2008JC005092

[CR59] Šepić J, Vilibić I, Lafon A et al (2015a) High-frequency sea level oscillations in the Mediterranean and their connection to synoptic patterns. Prog Oceanogr 137:284–298. 10.1016/j.pocean.2015.07.005

[CR60] Šepić J, Vilibić I, Fine I (2015b) Northern Adriatic meteorological tsunamis: Assessment of their potential through ocean modeling experiments. JGR Oceans 120:2993–3010. 10.1002/2015JC010795

[CR61] Šepić J, Vilibić I, Rabinovich AB, Monserrat S (2015c) Widespread tsunami-like waves of 23–27 June in the Mediterranean and Black Seas generated by high-altitude atmospheric forcing. Sci Rep 5:11682. 10.1038/srep11682 PMC4483776 26119833

[CR62] Šepić J, Međugorac I, Janeković I, Dunić N, Vilibić I (2016) Multi-meteotsunami event in the Adriatic Sea generated by atmospheric disturbances of 25–26 June 2014. Pure Appl Geophys 173:4117–4138. 10.1007/s00024-016-1249-4

[CR54] Skamarock WC, Klemp JB, Dudhia J, Gill DO, Barker DM, Duda MG, Huang X-Y, Wang W, Powers JG (2008) A description of the advanced research WRF version 3. UCAR/NCAR

[CR55] Stockdon HF, Holman RA, Howd PA, Sallenger AH (2006) Empirical parameterization of setup, swash, and runup. Coastal Eng 53:573–588. 10.1016/j.coastaleng.2005.12.005

[CR56] Suursaar Ü, Kullas T, Otsmann M, Saaremäe I, Kuik J, Merilain M (2006) Cyclone Gudrun in January 2005 and modelling its hydrodynamic consequences in the Estonian coastal waters. Boreal Env Res 11:143–159

[CR63] Thomson RE, Emery WJ (2024) Data Analysis Methods in Physical Oceanography, 4rd edn. Elsevier, Amsterdam Boston Heidelberg [etc.]

[CR65] Umgiesser G, Bajo M, Ferrarin C, Cucco A, Lionello P, Zanchettin D, Papa A, Tosoni A, Ferla M, Coraci E, Morucci S, Crosato F, Bonometto A, Valentini A, Orlić M, Haigh ID, Nielsen JW, Bertin X, Fortunato AB, Pérez Gómez B, Alvarez Fanjul E, Paradis D, Jourdan D, Pasquet A, Mourre B, Tintoré J, Nicholls RJ (2021) The prediction of floods in Venice: methods, models and uncertainty (review article). Nat Hazards Earth Syst Sci 21:2679–2704. 10.5194/nhess-21-2679-2021

[CR66] Vilibić I, Šepić J (2009) Destructive meteotsunamis along the eastern Adriatic coast: Overview. Phys Chem Earth 34:904–917. 10.1016/j.pce.2009.08.004

[CR67] Vilibić I, Šepić J (2017) Global mapping of nonseismic sea level oscillations at tsunami timescales. Sci Rep 7:40818. 10.1038/srep40818 PMC5241683 28098195

[CR68] Vilibić I, Šepić J, Pasarić M, Orlić M (2017) The Adriatic Sea: A Long-Standing Laboratory for Sea Level Studies. Pure Appl Geophys 174:3765–3811. 10.1007/s00024-017-1625-8

[CR69] Vilibić I, Rabinovich AB, Anderson EJ (2021) Special issue on the global perspective on meteotsunami science: editorial. Nat Hazards 106:1087–1104. 10.1007/s11069-021-04679-9

[CR70] Vilibić I, Zemunik Selak P, Šepić J (2025) Meteorological Tsunamis: from local hazard to global relevance. Rev Geophys. 10.1029/2024RG000867

[CR71] von Storch H, Woth K (2008) Storm surges: perspectives and options. Sustain Sci 3:33–43. 10.1007/s11625-008-0044-2

[CR72] Wunsch C, Stammer D (1997) Atmospheric loading and the oceanic inverted barometer effect. Rev Geophys 35:79–107. 10.1029/96RG03037

[CR73] Zängl G (2002) An Improved Method for Computing Horizontal Diffusion in a Sigma-Coordinate Model and Its Application to Simulations over Mountainous Topography. Mon Wea Rev 130:1423–1432.

[CR74] Zhang YJ (2014) SCHISM theory manual. Virginia Institute of Marine Science

[CR75] Zhang YJ, Ye F, Stanev EV, Grashorn S (2016) Seamless cross-scale modeling with SCHISM. Ocean Modell 102:64–81. 10.1016/j.ocemod.2016.05.002

